# Untargeted Metabolomics Reveals Raw Material Geographic Origin as a Key Factor Shaping the Quality of Ginger-Derived Exosome-like Nanovesicles

**DOI:** 10.3390/foods15020408

**Published:** 2026-01-22

**Authors:** Zhuo Chen, Xinyi Zhang, Liuliu Luo, Qiang Liu, Pingduo Chen, Jinnian Peng, Fangfang Min, Yunpeng Shen, Jingjing Li, Yongning Wu, Hongbing Chen

**Affiliations:** 1 State Key Laboratory of Food Science and Resources, Nanchang University, Nanchang 330047, China; 352335920004@email.ncu.edu.cn (Z.C.); 407900240102@email.ncu.edu.cn (X.Z.); 407900240074@ncu.edu.cn (L.L.); 417900230224@ncu.edu.cn (P.C.); min2fang@ncu.edu.cn (F.M.); syp@ncu.edu.cn (Y.S.); 357900230012@email.ncu.edu.cn (J.L.); 2 School of Food Science and Technology, Nanchang University, Nanchang 330031, China; 3 School of Pharmacy, Gannan Medical University, Ganzhou 341000, China; pengjinnian2017@gmu.edu.cn; 4 Sino-German Joint Research Institute, Nanchang University, Nanchang 330047, China; andyliu@dlut.edu.cn; 5 Faculty of Medicine, Dalian University of Technology, Dalian 116024, China; 6 NHC Key Lab of Food Safety Risk Assessment, China National Center for Food Safety Risk Assessment (CFSA), Beijing 100022, China; 7 Jiangxi Province Key Laboratory of Food Allergy, Nanchang University, Nanchang 330047, China

**Keywords:** food-derived nanomaterial, untargeted metabolomics, antioxidant activity, ginger, GELNs

## Abstract

A major challenge for food-derived bio-nanomaterials is achieving consistent and predictable functional properties to ensure their quality. Ginger-derived exosome-like nanovesicles (GELNs) serve as an ideal model for this challenge, yet the impact of ginger geographical origin on GELNs remains unknown. This study aims to establish a quality control framework for food-derived bio-nanomaterials. GELNs were comprehensively analyzed. Untargeted metabolomics identified differential metabolites, which were then screened for correlation with antioxidant capacity. Machine learning was employed to pinpoint potential quality markers, and Kyoto Encyclopedia of Genes and Genomes enrichment analysis highlighted key metabolic pathways. Significant variations in physicochemical properties and bioactivities were observed. We identified 190 differential compounds and established a panel of 6 potential quality markers. Enrichment analysis revealed eight key pathways, with “microbial metabolism in diverse environments” and “galactose metabolism” being most prominent. The quality marker mollicellin I (derived from *Chaetomium brasiliense*) provided empirical support linking GELNs quality to geography-specific microbiota. Our findings provide evidence that the geographic origin of raw materials is a primary determinant of GELNs quality, based on a systematic analysis of their chemical and functional properties. We develop a transferable quality control framework, laying the groundwork for producing superior natural food-derived nanomaterials.

## 1. Introduction

Ginger (*Zingiber officinale* Roscoe) is a high-value global commodity, with raw products contributing ~USD 4.69 billion annually [1,2], valued for its dual role as both a culinary agent and a health-promoting food [3,4,5]. There is a growing demand to precisely and safely harness ginger’s bioactive components for enhanced health benefits [6]. Plant-derived exosome-like nanovesicles (PELNs), known as plant-derived exosome-like nanoparticles, are edible nanomaterials, typically ranging from 30 to 300 nm in diameter [7]. Those originating from edible plants function as green nano-delivery platforms, transporting bio-actives to specific organs or cells to improve health [5,7]. As typical natural nanomaterials, ginger-derived exosome-like nanovesicles (GELNs) contain bioactive components such as proteins, lipids, gingerols, shogaols, and miRNAs, and similarly facilitate the targeted delivery of these substances [7,8]. These nanovesicles have shown antioxidant and anti-inflammatory effects, modulation of microbiota, prevention of insulin resistance, and anticancer properties [7,8,9]. These representative features and benefits indicate that GELNs can be used as a model material for studying the quality of edible nanomaterials from nature.

Implementing precise quality control for nanomaterials is essential for ensuring the reproducibility of their properties and functions [10,11]. However, achieving this for edible plant-derived exosome-like nanoparticles (PELNs) remains technically challenging [11,12]. A central obstacle lies in the lack of universal standardization markers. Unlike mammalian exosomes, which have conserved protein markers such as CD9 and TSG101, PELNs lack widely accepted protein standards [13,14,15]. While candidates like TET8 and PEN1 have been proposed, their utility remains contentious, with studies in *Arabidopsis thaliana*, for example, indicating that TET8 aligns with exosome profiles, whereas PEN1 does not [12,13]. Recent studies have identified another set of proteins as markers for GELNs, which also highlights the current lack of a standardized identification system [9]. Moreover, GELNs also exhibit heterogeneous microRNA profiles. For instance, osa-miR164d alleviates intestinal inflammation, and aly-miR159a-3p enhances anti-PD-L1 melanoma therapy [8,16]. Current analytical approaches for GELNs are yet to be optimized for food-grade quality control, a critical gap that necessitates multi-faceted investigations to define precise chemical quality control parameters and construct a holistic framework to underpin the practical application and credibility of these nanomaterials.

The chemical composition of a plant is influenced by its geographical origin, which includes genetics, environmental conditions, and agricultural practices [17,18,19]. Recent research on standardizing GELNs has emphasized cross-taxon comparative analyses to identify key functional components [9,20]. In contrast, while quality authentication based on geographic origin is well-established for ginger rhizomes using tissue-specific markers, it remains untested for GELNs due to their distinct composition [19]. It is therefore critical to determine whether geographic origin is a defining factor for the GELN profile, thereby establishing these markers as indicators of GELN quality.

Untargeted metabolomics utilizing liquid chromatography–mass spectrometry (LC-MS) represents the ideal analytical platform to test the hypothesis that geographic origin defines the PELNs profile [21]. This method has been extensively employed for comprehensive phytochemical profiling, demonstrated by its elucidation of underexplored chemical diversity in processed plant-derived protein-rich foods [22]. LC-MS-based untargeted metabolomics has been applied for establishing ginger metabolite libraries, identifying exposure biomarkers, and mapping chemical profiles [21,23]. Further, it can detect trace contaminants, a capability validated by its application in tracking plant pesticide residues [24]. However, the application of untargeted metabolomics to define the small-molecule composition of GELNs for identifying markers is currently lacking [8,9,16]. Identifying geographically related bioactive metabolites and contaminant markers of GELNs will fill gaps in their function and safety parameters and extend their marker identification paradigm to PELNs.

To determine how ginger’s geographic origin influences GELNs, we collected samples from three major ginger-producing regions in China: Xinguo County (Ganzhou City, Jiangxi Province; cultivation area: 3.33 × 10^6^ m^2^; annual output: ~5.0 × 10^6^ kg; annual production value: ~USD 7.136 million) [25], Bo’ai County (Henan Province; 3.33 × 10^6^ m^2^; annual output: >1.25 × 10^7^ kg; production value: >USD 37.1 million) [26], and Chaling County (Hunan Province; 3.33 × 10^6^ m^2^; annual output: ~5.0 × 10^6^ kg) [27]. The standardized cultivation area across these geographically distinct locations ensures a reliable supply of raw materials for the study. GELNs were then extracted from these samples, and a comparative analysis was performed on their core physicochemical traits (size distribution, surface charge, and chemical profiling) and functional bioactivities. Untargeted metabolomics was employed to comprehensively profile the small-molecule compositions. Partial least squares discriminant analysis (PLS-DA) and orthogonal partial least squares discriminant analysis (OPLS-DA) were utilized to characterize the heterogeneity. As antioxidant capacity serves as a pivotal index for formulating food quality standards and validating health claims [28,29], Caco-2 cell models were utilized to focus on evaluating this core functional property, while cellular internalization capacity was examined for auxiliary verification. Subsequent analyses included the identification of antioxidant-associated compounds through OPLS-DA and Spearman correlation analysis, with further validation implemented via random forest classification. Kyoto Encyclopedia of Genes and Genomes (KEGG) pathway analysis was conducted to elucidate the metabolic pathway variations associated with raw material geographic origin differences. This study aims to establish a quality control framework for food-derived biological nanomaterials by identifying key analytical parameters and elucidating the underlying mechanisms of quality variation. The objective is to trace the quality evolution of these materials throughout the entire supply chain, from farm to functional food products, thereby ensuring the reliability of their quality.

## 2. Materials and Methods

### 2.1. Plant Raw Materials and Reagents

The ginger samples for this study were collected from three major producing regions in China: Xinguo county-town in Ganzhou city of Jiangxi province (named Jiushan ginger, JS), Boai county-town in Henan province (named Boai ginger, BA), and Chaling county-town in Hunan province (named Chaling ginger, CL). The three ginger types were authentic regional products with both official and academic documentation of their origins, representing distinct geographical regions. In November 2023, fresh ginger rhizomes were harvested from core production areas in each region. Three independent batches (2 kg each) were purchased directly from different local farmers in each region to ensure representativeness. Key geographical features (climate, hydrothermal conditions, and soil properties) of the three origins are summarized in Supplementary Table S1, while their distinct morphological characteristics are shown in Figure S1. The fresh rhizomes were transported to the laboratory at ambient temperature using the fastest available logistics to minimize transit time. Upon arrival at our laboratory, they were stored under controlled conditions (13–15 °C, 90–95% RH) to preserve freshness until processing.

All chemicals, biological reagents, and cell lines were obtained as specified in the supporting files. Methanol and ammonium hydroxide, both of LC-MS grade, were procured from Fisher Scientific (Fair Lawn, NJ, USA). 2,2-di(4-tert-octylphenyl)-1-picryl-hydrazyl (DPPH), hydrogen peroxide (3%), 4′,6-diamidino-2-phenylindole (DAPI), and ammonium acetate (LC-MS grade) were acquired from Sigma-Aldrich (St. Louis, MO, USA). Acetonitrile of LC-MSgrade was obtained from Merck (Darmstadt, Germany). PBS (10 mM, pH 7.2–7.4) was purchased from Solarbio (Beijing, China). PBS × 1 was obtained from Cellmax (Seongnam, Republic of Korea). Additionally, 200-mesh copper grids with Formvar film were sourced from Zhongjing Keyi (Beijing, China). Uranyl acetate was purchased from Electron Microscopy Sciences (Hatfield, PA, USA). 2,2-azinobis (3-ethylbenothiazoline-6-sulfonic acid) (ABTS) and H2DCFDA (DCFH-DA) were provided by MedChemExpress (Monmouth Junction, NJ, USA). The Caco-2 cell line used in this study was originally purchased from the Kunming Cell Bank, Chinese Academy of Sciences (Kunming, China) and has been routinely maintained in our laboratory. CCK-8 was purchased from APExBIO (Houston, TX, USA). The bicinchoninic acid (BCA) protein quantification assay kit was obtained from Biyuntian Biotechnology Co., Ltd. (Shanghai, China). Phalloidin-FITC was bought from Abcam (Cambridge, UK).

### 2.2. Isolation, Purification, and Characterization of GELNs

GELNs were isolated, purified, and characterized following the reported methods with certain modifications [30,31,32]. The improved extraction method was used to obtain GELNs with higher yield, purity, and minimized co-extracted extraneous compounds to ensure reliable downstream metabolomics analysis. Fresh ginger roots were peeled and cut into 1 cm^3^ pieces. Subsequently, 240× *g* of the processed material was immersed in 4 °C ice-cold phosphate-buffered saline (PBS; 10 mM, pH 7.2–7.4). After removing the PBS, the ginger was juiced using a slow juicer (Mokkom, Foshan, China). The ginger juice was centrifuged at 4 °C in a series of steps: First, centrifuge at 1000× *g* for 10 min and then at 4000× *g* for 20 min to remove debris and organelles. Next, centrifuge the supernatant at 10,000× *g* for 25 min and repeat with the resuspended pellet to obtain a crude GELNs prep. Filter the resulting supernatant through a 450 nm polyethersulfone membrane for the primary filtrate. Finally, ultracentrifuge 36 mL of the primary filtrate at 100,000× *g* for 2 h for purification. The resulting pellets were resuspended in 5.0 mL of cold PBS × 1 and passed through 450 nm and 200 nm polyethersulfone filters (Pall Life Sciences, Port Washington, MI, USA) in sequence. Nine GELNs samples were stored at −80 °C for subsequent analysis.

The particle size and number of GELNs were measured using a Zetaviewer (Particle Metrix, Inning am Ammersee, Germany) at fixed concentration, while zeta potential was determined with a NanoBrook 90Plus analyzer (Brookhaven Instruments Corp., Holtsville, NY, USA). The morphology of the GELNs was observed by transmission electron microscopy (TEM, JEM-1400, JEOL, Peabody, MA, USA). Briefly, 20 µL of resuspended GELNs was added to 200-mesh copper grids and incubated for 10 min. The GELNs were stained with 2% phosphotungstic acid for 5 min, blotted dry, and observed via TEM at 80 kV. The GELNs protein concentrations were quantified using a BCA protein assay kit.

### 2.3. The GELN Functional Assessment

The antioxidant capacity of GELNs was assessed by measuring radical scavenging activity (DPPH and ABTS^·+^ assays), quantifying intracellular reactive oxygen species (ROS) levels, and evaluating oxidative stress resistance. Moreover, the effect of GELNs on cell viability was analyzed, and their cellular internalization was observed.

#### 2.3.1. DPPH and ABTS^·+^ Radical Scavenging Activities Assay

For the DPPH radical scavenging activity assay, a stable 100.48 µM DPPH solution was prepared in methanol [33,34]. This solution was then mixed with 20 µL of the GELN sample (1.0 mg/mL) in a test tube, and the absorbance was measured at 517 nm at 30 min intervals using a microplate reader (Varioskan Flash^TM^, Thermo, Waltham, MA, USA). For the ABTS^·+^ radical scavenging activity assay, the ABTS^·+^ radical cation was generated by incubating a 7 mM ABTS stock solution with 2.45 mM potassium persulfate (K_2_S_2_O_8_) for 15 h in the dark. After adjusting the absorbance of the ABTS solution to 0.70 ± 0.02 at pH 7.4 using PBS, 20 µL of the prepared sample was mixed with 275 µL of the ABTS solution. The absorbance was measured after 6 min (plateau phase) in the dark at 734 nm using a microplate reader (Varioskan Flash^TM^, Thermo, USA).

The percentage of DPPH and ABTS^·+^ scavenging activity was calculated as % inhibition using the following Equation (1):

Inhibition (%) = (A_1_ − A_0_)/A_1_ × 100% (1)

where A_0_ is the mean absorbance of the sample, and A_1_ is the mean absorbance of the control.

#### 2.3.2. Cell Culture and Cell Viability

As described, Caco-2 cells were seeded onto 96-well plates at a density of 5 × 10^3^ cells per well and incubated at 37 °C for 24 h. Cell viability was assessed using the CCK-8 assay [35,36]. GELNs at concentrations of 0, 60, 120, and 240 µg/mL (100 µL per well) were added and incubated for 24 h, with non-treated cells serving as the negative control (NEGc). A 10% CCK-8 solution was then added and incubated for 1 h, after which absorbance was measured at 450 nm using a Varioskan Flash^TM^ microplate reader (Thermo, USA). Cell viability was calculated according to the following Equation (2):

Caco-2 cell viability (%) = (A_sample_ − A_blank_)/(A_NEGc_ − A_blank_) × 100% (2)

where A_Sample_ is the mean absorbance of wells containing cells, culture medium, CCK-8 solution, and sample (Samples); A_NEGc_ is the mean absorbance of wells containing cells, culture medium, and CCK-8 solution (negative control); and A_Blank_ is the absorbance of wells containing culture medium and CCK-8 solution but no cells (blank control).

#### 2.3.3. Intracellular ROS Measurement

The antioxidant activity of GELNs was assessed in Caco-2 cells using the fluorogenic probe DCFH-DA, following the described protocols [35,36]. Briefly, Caco-2 cells were seeded in 96-well plates at 5 × 10^3^ cells per well and incubated for 24 h. The medium was then replaced with GELNs at concentrations of 0, 60, 120, and 240 µg/mL. After 24 h, DCFH-DA (10 µM final concentration) was added and incubated for 30 min at 37 °C. To induce oxidative stress, a 1.75 mM H_2_O_2_ solution was added to the culture medium for 3 h prior to DCFH-DA treatment. Fluorescence was measured at excitation 485 nm and emission 528 nm using a Varioskan Flash^TM^ microplate reader (Thermo, USA). The results were expressed as a percentage of ROS levels relative to the negative control (NEGc), which consisted of cells treated with medium only, while the positive control (POSc) involved cells treated with H_2_O_2_ for 3 h. The ROS levels were calculated using the following Formula (3):

ROS level (%) = (A_sample_ or A_POSc_/A_NEGc_) × 100% (3)

where A_Sample_ is the average fluorescence intensity in wells containing cells, culture medium, DCFH-DA, and the sample (Samples group, cells treated with GELNs and H_2_O_2_). A_NEGc_ is the average fluorescence intensity in wells containing cells, culture medium, and DCFH-DA but no GELNs and H_2_O_2_ (negative control group, cells treated with neither GELNs nor H_2_O_2_). A_POSc_ is the average fluorescence intensity in wells containing cells, culture medium, and DCFH-DA but no GELNs, only H_2_O_2_ (positive control group, cells treated with only H_2_O_2_).

#### 2.3.4. Cellular Internalization Capacity Assay

GELNs were labeled with the fluorescent lipophilic dye PKH26, and their internalization capacity by Caco-2 cells was assessed [37]. Typically, GELNs (500 µL, 1 mg/mL) were labeled with PKH26 to a final concentration of 5 µM, followed by washing with PBS and ultra-high-speed centrifugation for 70 min at 4 °C. The pellets were resuspended in 200 µL of PBS. Caco-2 cells were seeded in 12-well plates at 1 × 10^5^ cells/well and incubated for 12 h. PKH26-labeled GELNs (0.24 mg/mL) from JS, QH, and CL gingers were added and incubated at 37 °C for 24 h. Cells were then fixed with 4% paraformaldehyde for 15 min. Subsequently, 100 µL of phalloidin-FITC (5 µg/mL) was added to the cells and incubated for an additional 60 min at room temperature. Following this, the cells were rinsed three times with phosphate-buffered saline containing 0.1% Tween-20 (PBST), with each wash lasting 3 min. Cell nuclei were then stained with 4′,6-diamidino-2-phenylindole (DAPI) for 5 min. Excess DAPI was removed by rinsing the cells four times with PBST, with each wash lasting 5 min. Finally, the cells were observed and imaged using a Leica SP8 confocal microscope (Leica Microsystems, Wetzlar, Germany) equipped with the LAS X Life Science Microscope Software Platform, version 4.5.0. The average fluorescence intensity was quantified using Fiji (ImageJ) software (version 1.53t; National Institutes of Health, Bethesda, MD, USA). Three random fields of view per sample were analyzed. The scale was calibrated using the 50 μm scale bar included in all images.

### 2.4. Metabolomic Samples and Analysis

Metabolomic samples were prepared and analyzed according to a previously described method [38]. Briefly, 100 μL of GELN samples was mixed with a precooled 2:2:1 methanol/acetonitrile/water solution, then sonicated at −20 °C for 30 min. After centrifugation at 14,000× *g* for 20 min, the supernatant was dried and redissolved in 100 μL of acetonitrile/water (1:1). The sample was then vortexed and centrifuged again at 14,000× *g* for 15 min, with the final supernatant serving as the sample solution to be tested. All the metabolite samples were mixed to become three quality control (QC) samples. The 2 μL samples were separated using a Vanquish UHPLC system and Q Exactive mass spectrometer (Thermo, USA) with data-dependent acquisition. The metabolites were separated using a 1.7 μm, 2.1 mm × 100 mm ACQUITY UPLC^®^ BEH Hilic column (Waters, Dublin, Ireland) at 25 °C with a flow rate of 0.3 mL/min. The mobile phase was composed of A (25.0 mM ammonium acetate/ammonia hydroxide in water) and B (acetonitrile). The gradient elution was as follows: 0–1.5 min, 98% B; 1.5–12 min, B decreased to 2%; 12–14 min, 2% B; 14–14.1 min, B increased to 98%; 14.1–17 min, 98% B. Samples were kept at 4 °C in an autosampler and analyzed in random order to avoid signal fluctuation. The Q Exactive mass spectrometer was used with electrospray ionization (ESI) settings: nebulizer gas 1 and auxiliary gas 2 at 60 psi, air curtain at 30 psi, source temperature at 600 °C, and spray voltage at ±5.5 kV. Primary MS detection covered 80–1200 Da with a 60,000 resolution and 100 ms scan time. Secondary MS used a segmented method, scanning 70–1200 Da with a 30,000 resolution, 50 ms scan time, and 4 s dynamic exclusion. QC samples were strategically interspersed within the sample cohort to monitor the stability and reliability of the data. The metabolomic data obtained from both ESI (+) and ESI (−) modes was integrated for subsequent analysis, following the outlined procedure [39]. The raw mass spectrometry data were converted to mzXmL format using Proteo Wizard (v3.0.8789). Peak detection, retention time alignment, and intensity quantification were conducted using the XCMS package in R (version 3.1.3). For confident annotation of metabolites, a mass accuracy threshold of ≤25 ppm was established. Metabolite identification was performed by matching experimentally acquired high-resolution MS/MS spectra against a combination of in-house (Wekemo Technology Group Co., Ltd. in Shenzhen, China) and public spectral databases, including Human Metabolome Database (https://www.hmdb.ca/, accessed on 30 January 2024), MassBank (https://massbank.eu/, accessed on 30 January 2024), MetLin (https://metlin.scripps.edu/, accessed on 30 January 2024), and MoNA (https://mona.fiehnlab.ucdavis.edu/, accessed on 30 January 2024). Spectral matches were assigned similarity scores on a scale from 0 to 1, where 1 indicates a perfect match. Annotation confidence followed Level 2 criteria (putative annotation) as established by the Metabolomics Standards Initiative. Under these guidelines, 98.4% of annotated compounds displayed spectral matching scores exceeding 0.7, with the remainder achieving scores no lower than 0.62. Peak intensities were normalized relative to the total spectral intensity and sample protein concentration. The data were analyzed using MetaboAnalystR packages 2.0.1 in R, as well as the Metware Cloud platform (https://cloud.metware.cn, accessed on 24 August 2025). The OPLS-DA model, which has the ability to more effectively discriminate between-group differences, was employed to identify key differential compounds based on a Variable Importance in the Projection (VIP) value > 1 and a *p* < 0.05 [28,40]. Spearman correlation analysis (|ρ| > 0.7, *p* < 0.05) was performed in R to assess relationships between untargeted metabolomic profiles and antioxidant activity in GELNs [18]. Random forest modeling validated the potential quality markers based on their maximal contribution to variation [28]. Different metabolites were annotated with KEGG (*p* < 0.05) and enriched pathways identified via over-representation analysis (ORA) (FDR *p* < 0.05) [41,42].

### 2.5. Statistical Analysis

Data from three independent experimental replicates (n = 3) were expressed as the mean ± SD. For statistical analysis, a one-way analysis of variance (ANOVA) was performed, followed by the least significant difference (LSD) post hoc test using GraphPad Prism (v10.2.3). Differences were considered statistically significant at *p* < 0.05.

## 3. Results

### 3.1. Characterization

**Figure 1:**
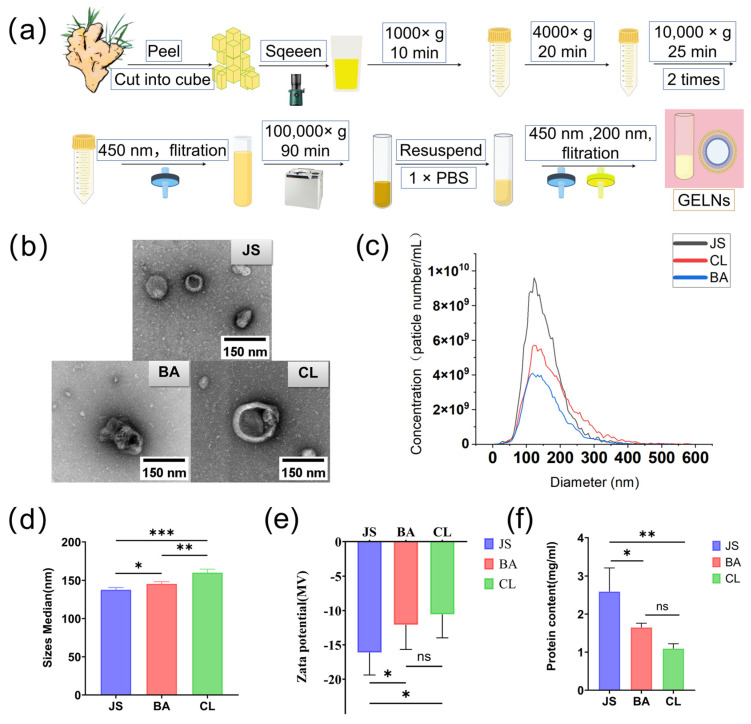
Characteristics of ginger-derived extracellular-like vesicles (GELNs) from gingers sourced from three distinct geographic locations. (**a**) Workflow for the extraction of GELNs. (**b**) Morphology of GELNs. Scale bars denote 150 nm. (**c**) Particle number distribution of GELNs (n = 3). (**d**) Median particle size of GELNs (n = 3). (**e**) Surface charge of GELNs (n = 3). (**f**) Protein concentration of GELNs extracted from an equal volume of ginger juice (n = 3). Data are mean ± SD. An asterisk (*) is used to denote statistical significance at *p* < 0.05, a double asterisk (**) is employed to indicate significance at *p* < 0.01, and a triple asterisk (***) is utilized to signify significance at *p* < 0.001; ns indicates no significance (*p* > 0.05).

As shown in Figure 1a, the GELNs were extracted by using the differential centrifugation method combined with the filtration method. TEM analysis confirmed that a classic cup-shaped morphology analogous to exosomes was displayed by all isolated GELNs, yet with quantifiable ultrastructural distinctions (Figure 1b). The three types of GELNs with varying concentrations per milligram of protein were as follows: (2.0 ± 0.1) × 10^11^ particles/mL (JS), (1.0 ± 0.0) × 10^11^ particles/mL (BA), and (1.6 ± 0.0) × 10^11^ particles/mL (CL) (Figure 1c). Distinct particle sizes were further shown by them: the size of JS was 137.73 ± 2.32 nm, the size of BA was 145.43 ± 2.38 nm, and the size of CL was 160.13 ± 3.70 nm (Figure 1d). Differential negative zeta potential values among the GELNs were revealed by surface charge analysis, with the order CL > JS > BA (Figure 1e). Additionally, different protein concentrations were displayed by the GELNs from three gingers of different geographic origins under the same extraction method (Figure 1f).

### 3.2. Multivariate Statistical Analysis

The total ion chromatograms (TICs) of QC samples in both ion modes show consistent intensities and retention times, indicating the stability and reliability of the data (Figure S2). A total of 1272 compounds were identified in all GELNs derived from three geographically distinct gingers. Based on chemical taxonomy, they were classified into 15 superclasses and 91 classes. The results demonstrated that all GELNs possess diverse and complex chemical compositions, as illustrated by the superclass distribution, top 20 chemical classes, and the 50 most abundant compounds presented in Figure S3.

**Figure 2:**
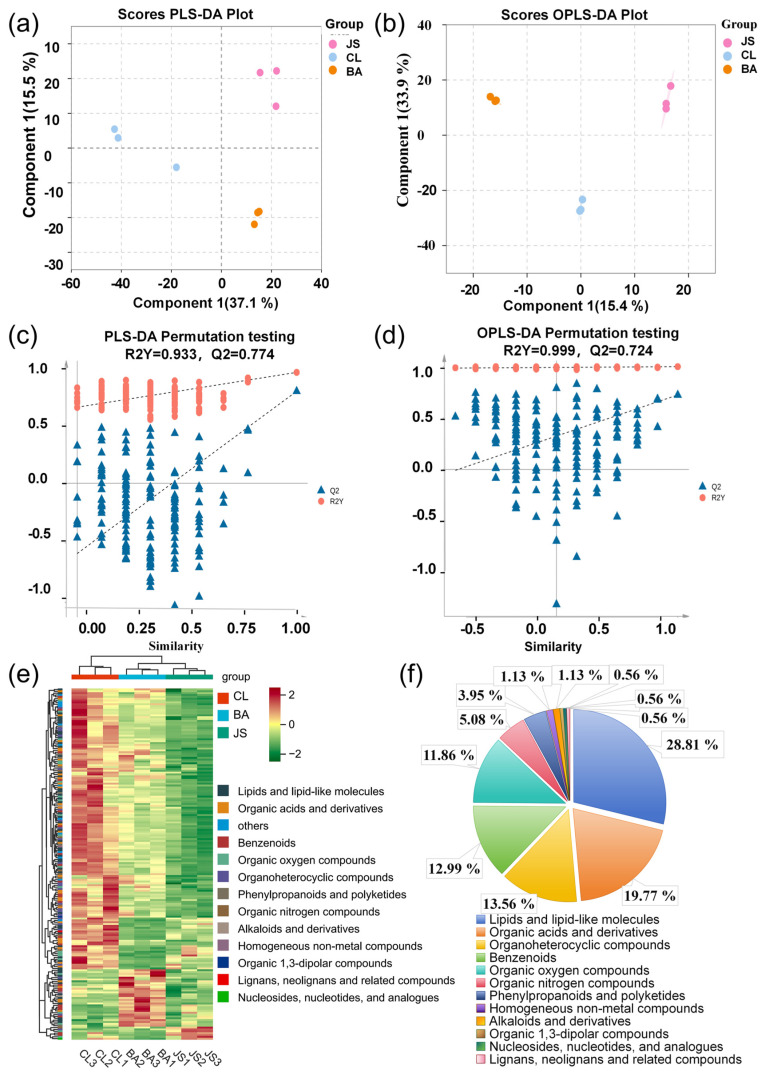
Results of PLS-DA and OPLS-DA of compounds in GELNs sourced from gingers in three locations, and key differential component analysis. (**a**) PLS-DA score plots. (**b**) OPLS-DA score plots. (**c**) PLS-DA permutation test plot (200 iterations). Q2 = (0.0, −0.589). (**d**) OPLS-DA permutation test plot (200 iterations). Q2 = (0.0, 0.324). (**e**) Proportions of key different compounds in the superclass of GELNs from gingers of three geographic locations. (**f**) Cluster analysis results of key different compounds in GELNs from gingers of three geographic locations (The percentages are based on the original calculations. The sum may not equal 100% due to rounding to two decimal places).

PLS-DA and OPLS-DA were used for the multivariate analysis of these compounds to analyze the differences among the three GELNs. In the PLS-DA model, the first two principal components, namely PC1 and PC2, accounted for 37.1% and 15.5% of the total variance, respectively, with a cumulative contribution of 52.6% to the variation (Figure 2a). Likewise, in the OPLS-DA model, PC1 and PC2 explained 15.4% and 33.9% of the variance, respectively, cumulatively accounting for 49.3% (Figure 2b). These findings clearly show that both the PLS-DA and OPLS-DA models can effectively distinguish GELN samples from different geographical origins. In the 200-time permutation tests, the predictive accuracy of the generated plots of these two models was confirmed by R2Y and Q2 (Figure 2c,d). Moreover, the *p*-values for Q2 in the permutation tests were less than 0.05, providing additional validation that the models were not overfitted (Figure S4a,b). The compounds identified were distinctly different and were effectively classified into three categories among the GELNs from the three locations.

### 3.3. Screening and Classification of Key Difference Composition

A total of 190 key different compounds (VIP > 1.0, *p* < 0.05) were identified in GELNs from three geographically distinct gingers (Table S3). The relative abundance data (presented as Mean ± SD) for all differential metabolites across the three GELN types are provided in Supplementary Table S3. Hierarchical cluster analysis (HCA), using the z-score for data standardization, successfully clustered the differential metabolites from the three ginger varieties (JS, BA, CL) into three distinct groups (Figure 2e). Among these, 177 key differential compounds were classified into 12 superclasses, including lipids and lipid-like molecules, organic acids and derivatives, organoheterocyclic compounds, benzenoids, organic oxygen and nitrogen compounds, phenylpropanoids and polyketides, alkaloids and derivatives, homogeneous non-metal compounds, lignans, neolignans and related compounds, nucleosides, nucleotides, and analogues, and organic 1,3-dipolar compounds (Figure 2f). The top superclass, lipids and lipid-like molecules, encompasses 51 compounds, which account for over a quarter of the key differential metabolites. Cluster analysis successfully categorized lipids from three different ginger origins, and their composition and clustering patterns exhibited similarities to those observed in different compounds (Figure S5).

### 3.4. The Result of Functional Assessment

To explore the heterogeneity of bioactivity of GELNs from three different ginger origins, the effects of GELNs on Caco-2 cells viability, antioxidant capacity, and the internalization of GELNs by Caco-2 cells were compared.

**Figure 3:**
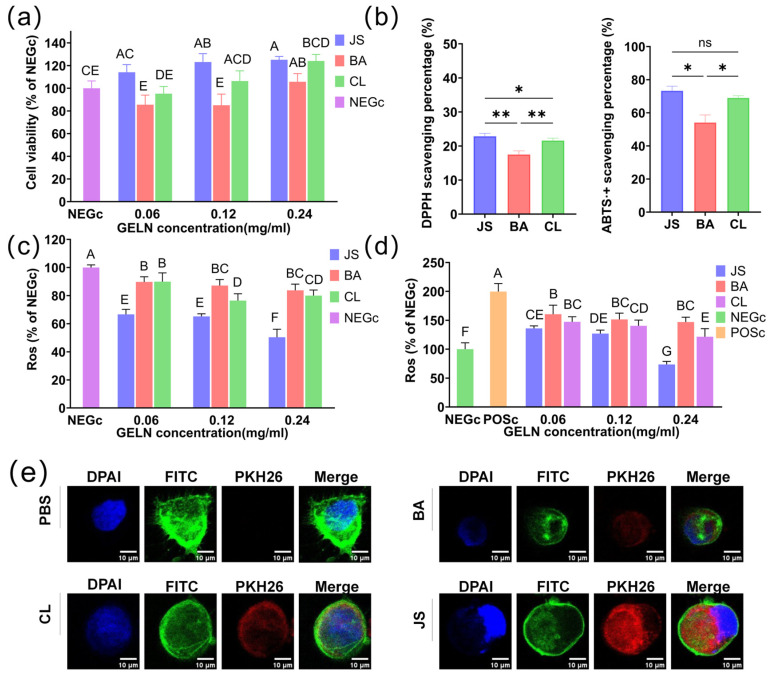
Antioxidant properties of GELNs from three ginger varieties. (**a**) The effect of GELNs on the viability of Caco-2 cells (n = 3). (**b**) The DPPH and ABTS^·+^ radical scavenging activities of GELNs (n = 3). (**c**) The levels of reactive oxygen species (ROS) in Caco-2 cells treated with GELNs (n = 3). (**d**) The ROS levels in Caco-2 cells pretreated with GELNs and then treated with H_2_O_2_ (n = 3). (**e**) Representative confocal micrographs of Caco-2 cells showing the internalization of PKH26-labeled GELNs (red) from the three geographical origins (BA, CL, JS) and a negative control. Scale bars represent 10 μm. Data are mean ± SD. An asterisk (*) is used to denote statistical significance at *p* < 0.05, a double asterisk (**) is employed to indicate significance at *p* < 0.01, while ns indicates no significance (*p* > 0.05). Identical letters represent no significant differences (*p* > 0.05) as determined by the compact letter display (CLD) method.

The viability of Caco-2 cells treated with GELNs exhibited a dose-dependent increasing trend. Specifically, at a concentration of 0.24 mg/mL, the viability of JS reached 125.16 ± 2.87%, surpassing that of CL (124.10 ± 5.75%) and BA (105.72 ± 7.18%) (Figure 3a).

The antioxidant properties of GELNs derived from three different ginger origins (JS, CL, BA) were evaluated through multiple assays, indicating that the GELNs exhibit varying levels of antioxidant capacity. In DPPH radical scavenging assays, JS GELNs demonstrated significantly higher activity than both BA (*p* < 0.01) and CL (*p* < 0.05) GELNs, while CL GELNs also outperformed BA GELNs (*p* < 0.01) (Figure 3b). ABTS^·+^ scavenging activity showed no significant differences among most varieties, although BA GELNs exhibited markedly lower activity than CL (*p* < 0.01) (Figure 3b). Notably, JS GELNs displayed a 19.17% higher scavenging rate than BA (*p* < 0.001), further highlighting the antioxidant superiority of JS. All GELNs significantly reduced basal ROS levels across the tested concentration range (0.06–0.24 mg/mL; *p* < 0.05) (Figure 3c). At 0.12 mg/mL, distinct ROS reduction efficiencies emerged among the varieties, with JS GELNs demonstrating superior activity compared to CL and BA (*p* < 0.05). At concentrations of 0.06 mg/mL and 0.24 mg/mL, no significant disparities were observed in the ROS-reducing abilities between CL and BA GELNs (*p* > 0.05). Under H_2_O_2_-triggered stress, all GELNs showed antioxidant stress function by reducing ROS (Figure 3d). At 0.24 mg/mL, statistical analysis showed significant differences (*p* < 0.05) in ROS reduction capacities among the three GELNs.

The internalization of PKH26-labeled GELNs (0.24 mg/mL) in Caco-2 cells was qualitatively analyzed by confocal fluorescence microscopy. Visual comparison revealed apparent differences in signal distribution, with JS GELNs showing more extensive intracellular fluorescence coverage and greater relative intensity compared to CL and BA GELNs (Figure 3e and Figure S6), suggesting a trend in internalization efficiency of JS > CL > BA. This trend was supported by quantitative analysis, which showed that the average fluorescence intensity of JS GELNs (43.92 ± 35.40 a.u.) was significantly higher than that of both CL (26.66 ± 14.68 a.u.) and BA GELNs (24.70 ± 13.93 a.u.). These statistical results confirm that the superiority of JS GELNs is significant, while the difference between CL and BA GELNs is not statistically significant. These research findings suggest a systematic association between the geographical origin of ginger and the cellular internalization behavior of its GELNs.

### 3.5. Relationship Between Differential Compounds and Antioxidant Ability

**Figure 4:**
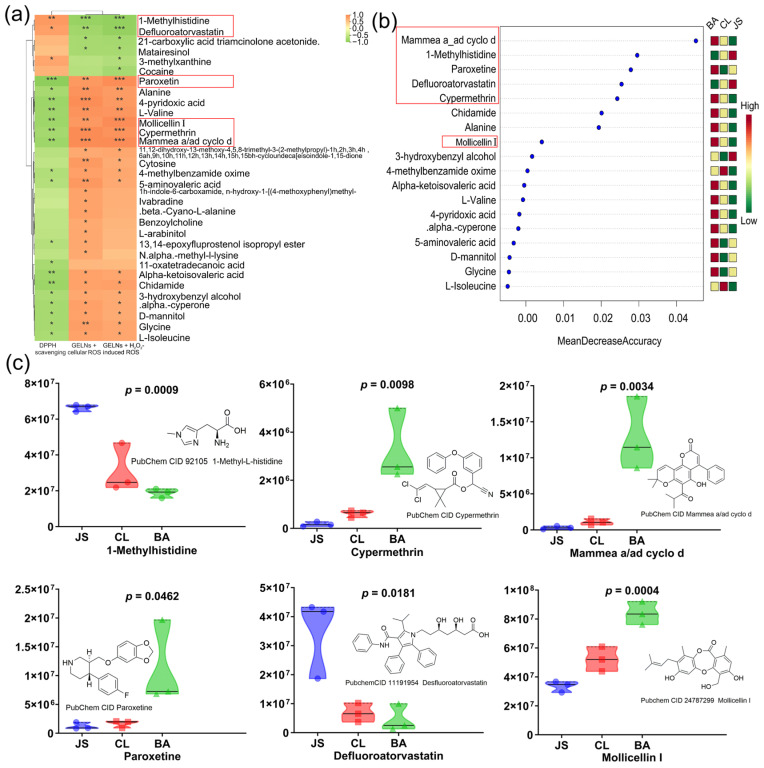
The potential quality marker of GELNs. (**a**) Results of the Spearman analysis of the correlation between differential compounds and antioxidant activity in three GELNs. Potential quality markers are indicated by the red box. (**b**) Results of the random forest model used to identify the compounds that contribute most to antioxidant activity. Potential quality markers are indicated by the red box. (**c**) Diversity of six potential quality markers in GELNs from ginger of three geographic origins. An asterisk (*) is used to denote statistical significance at *p* < 0.05, a double asterisk (**) is employed to indicate significance at *p* < 0.01, and a triple asterisk (***) is utilized to signify significance at *p* < 0.001.

To explore the relationship, a Spearman analysis of the different compounds of GELNs from gingers of various locations and their antioxidant abilities was conducted. A total of 6 compounds showed strong positive correlations (ρ > 0.7, *p* < 0.05) with GELN antioxidant capacity, while 26 compounds exhibited significant negative correlations (ρ < −0.7, *p* < 0.05) (Figure 4a). The compounds were characterized by the highest VIP values in the OPLS-DA model (Table S3). A total of 18 compounds were related to the three assessed antioxidant activities (Figure 4a). Screening of the compounds using the random forest model revealed an overlap of six compounds in the top 10 rankings between the two models (Figure 4b and Table 1). Four components that are negatively correlated with antioxidant activity (mollicellin I, cypermethrin, mammea a/ad cyclo d, paroxetine) and two components with positive correlations (1-methylhistidine, defluoroatorvastatin) were identified as potential quality markers (Figure 4b and Table 1). As shown in Figure 4c, the JS GELN samples exhibited the strongest antioxidant capacity, characterized by the highest contents of 1-methylhistidine and defluoroatorvastatin, while the contents of mollicellin I, cypermethrin, mammea a/ad cyclo d, and paroxetine were the lowest. In contrast, the BA GELN samples with the weakest antioxidant capacity had the opposite content distribution of these six compounds compared to the JS GELN samples. The antioxidant activity of the CL GELNs was at an intermediate level, and the content levels of these six compounds in their samples were between those of the BA and JS samples.

### 3.6. KEGG Pathway Analysis

**Figure 5:**
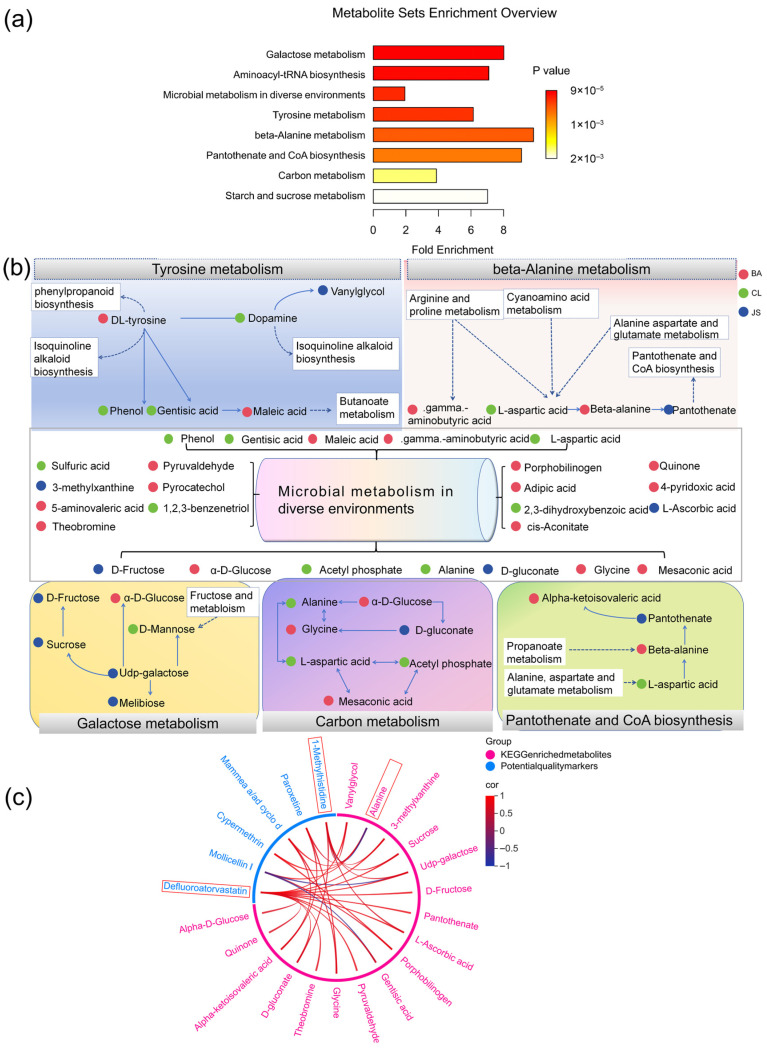
The result of KEGG metabolic pathway enrichment analysis. (**a**) The different metabolites were enriched in eight metabolic pathways in KEGG. (**b**) Metabolites enriched in each pathway are grouped within the same box. The red, green, and blue dots indicate that the substances in the BA GELN, CL GELN, and JS GELN groups exhibited maximum expression, respectively. White boxes represent other interacting metabolic pathways. (**c**) The relationship between KEGG enriched metabolites and potential quality markers (|r| > 0.6, *p* < 0.05).

763 compounds were identified with KEGG IDs, among which 201 showed significant differential abundance (*p* < 0.05). Over-representation analysis (ORA) revealed eight significantly enriched metabolic pathways (FDR *p* < 0.05) (Figure 5a). These included two pathways directly involved in sugar metabolism: galactose metabolism (ko00052, 6 metabolites enriched) and starch and sucrose metabolism (ko00500, 4 metabolites enriched). A total of six key pathways were consistently detected across all GELN groups: galactose metabolism (ko00052), microbial metabolism in diverse environments (ko01120, 26 metabolites enriched), tyrosine metabolism (ko00350, 6 metabolites enriched), beta-alanine metabolism (ko00410, 4 metabolites enriched), pantothenate and CoA biosynthesis (ko00770, 4 metabolites enriched), and carbon metabolism (ko01200, 7 metabolites enriched). The specific enrichment of different metabolites in pathways is shown in Figure 5b. Several key compounds are simultaneously enriched in different metabolic pathways. For instance, C00049 was found to be enriched in four pathways, namely ko01120, ko00410, ko00770, and ko01200, while C00267 was implicated in three pathways: ko00052, ko01120, and ko01200.

Correlation analysis further demonstrated that 17 out of the 36 compounds enriched in these key pathways were significantly correlated (|r| > 0.6, *p* < 0.05) with potential quality markers (Figure 5c). Defluoroatorvastatin and 1-methylhistidine were identified as the most extensively correlated metabolites, exhibiting interactions with 14 and 7 metabolites, respectively. A cluster of six metabolites, vanylglycol, D-gluconate, L-ascorbic acid, UDP-galactose, sucrose, and 3-methylxanthine, were positively correlated with both 1-methylhistidine and defluoroatorvastatin. Furthermore, defluoroatorvastatin showed additional positive correlations with D-fructose, pantothenate, gentisic acid, pyruvaldehyde, theobromine, quinone, and alpha-D-glucose. Alanine emerged as a key hub metabolite, showing a positive correlation with mollicellin I, paroxetine, mammea a/ad cyclo d, and cypermethrin, while correlating negatively with 1-methylhistidine. Additional correlation patterns included positive relationships between alpha-ketoisovaleric acid/glycine and paroxetine/mammea a/ad cyclo d, as well as between porphobilinogen and mollicellin I/cypermethrin. The network also revealed significant negative correlations, particularly between mollicellin I and both UDP-galactose and gentisic acid. These findings elucidate the metabolic interactions between key pathways and quality-related components, providing a mechanistic basis for precise quality and safety control.

## 4. Discussion

In this study, GELNs were successfully isolated from gingers of distinct geographical origins. Our untargeted metabolomics analysis decisively revealed that the geographic origin of the raw material is an important factor shaping the metabolite profile of GELNs. This geographical imprint was further reflected in concomitant variations in their biological activities, confirming a direct link between origin-specific chemical composition and functionality. The differential metabolites and key metabolic pathways identified herein not only elucidate the mechanistic basis for these functional disparities but also establish a concrete chemical foundation for quality and safety evaluation. Collectively, these findings validate the initial establishment of an analytical framework that integrates metabolomics with functional assessment to trace the geographical origin and predict the functional quality of natural edible nanomaterials like GELNs.

### 4.1. Methodological Optimization in Extraction

Typically, the extraction methods of plant-derived exosome-like nanovesicles predominantly rely on density and particle size [7,14,43]. Classical extraction uses differential ultracentrifugation with a sucrose density gradient, while alternative methods use PEG600-based polymer precipitation [14,44]. All of these methods introduce exogenous substances, which are detrimental to the study of the biogenesis of plant-derived exosome-like nanovesicles. For instance, the introduction of sucrose, a substance produced by most plants, can obscure the intrinsic physiological metabolism of plants. The enriched sugar metabolism pathways in this study further confirmed that sucrose-based isolation and purification may adversely affect GELN biogenesis research. In the early days, certain researchers successfully isolated ginger-derived exosome-like nanovesicles based on particle size [14,30]. Hence, in this study, based on this extraction method, the sequence of filtration and ultracentrifugation was optimized, followed by extraction. Characterization using TEM, the gold-standard nanoparticle tracking analysis (NTA) method, and zeta potential detection demonstrated that a large number of GELNs with a double-membrane structure, an optimal particle size of approximately 130–160 nm, and a negative charge were successfully extracted. In this study, an improved extraction method is not only efficient but also avoids the introduction of foreign components, which is more conducive to the study of the biogenesis of natural nanomaterials.

### 4.2. Intraspecific Comparison and Quality Parameters

Previous research has demonstrated that ginger sourced from diverse geographical locations can be traced through specific components [19,45]. The question of whether regional differences in ginger lead to variations in the quality of ginger exosome-like nanovesicles remains inadequately investigated. A recent study by Wang et al. demonstrated that the functional lipid composition of PELNs within the same genus is relatively similar, while the lipid components differ more significantly between different families [9]. Consequently, the comparison of PELNs within a species can rule out those components that are substantially influenced by interspecies variations. Furthermore, this study demonstrates that GELN yield can serve as a key indicator for evaluating the quality of these nanovesicles. This research validated the idea that comparing plants of the same species from different locations helps obtain more accurate quality-related parameters. It was a well-established fact that the biological functions of GELNs depended on components such as gingerols, which served as the active ingredients of ginger [9]. However, in this study a positive correlation between protein content and ginger’s active ingredients (6-gingerol, 8-gingerol, 10-gingerol) in GELNs of three varieties was observed through Pearson correlation analysis (|r| > 0.8, *p* < 0.05), and there were no significant differences at the unit protein concentration level (Figure S7). These active ingredients in ginger were unable to comprehensively account for the intraspecific quality variations of GELNs at the same protein concentration. At the same time, this also poses higher requirements for the precise evaluation of the quality and safety of GELNs.

### 4.3. A Framework for Screening Quality Markers

Antioxidant activity and non-target metabolomics screening has been described as a feasible approach for identifying potential quality markers [46]. In this investigation, three types of GELNs from different origins had antioxidant effects, but there were significant differences in their antioxidant potency (*p* < 0.05). Our results indicated that the internalization behavior of Caco-2 cells toward the three types of GELNs followed a similar trend, with JS exhibiting the highest internalization efficiency, while BA and CL showed comparable levels of uptake. This suggests that the antioxidant activity of GELNs aligns with the trend observed in their core functions. This further supports that combining antioxidant activity analysis with non-targeted metabolomics is an effective approach for identifying quality markers of GELNs. The metabolite signatures associated with geographical origin, as identified through rigorously validated models, should be interpreted with caution. While these associations have been cross-verified via random forest analysis, they remain correlative rather than indicative of causation. The functional relationships proposed in the following section are therefore presented as plausible biological hypotheses that warrant further investigation.

In our approach, six components affecting GELNs’ antioxidant strength were identified. Among the identified endogenous components, 1-methylhistidine and defluoroatorvastatin are potential markers with appropriate stability [47,48]. Mammea a/ad cyclo d was associated with the ROS-induced anticancer effect and is negatively correlated with antioxidant activity [49,50]. Therefore, these three endogenous components are proposed as potential quality markers. As the correlation analysis (Figure 5c) demonstrates, the geographic variation in the accumulation of ginger antioxidant metabolites within GELNs is possibly associated with contaminant-induced metabolic disruption. Early studies established that organic cultivation enhances the antioxidant capacity of plants; the specific impact of organic methods on the yield and antioxidant activity of GELNs remained unclear [51]. This study reveals an association between geographic origin and three main categories of contaminants in GELNs: mycotoxins, pesticide residues, and pharmaceutical contaminants [10]. Specifically, the presence of mollicellin I, a metabolite from the soil fungus *Chaetomium brasiliense*, serves as an indicator of fungal-derived contamination [52,53,54]. Cypermethrin, as a widely used insecticide, shows variation in residue levels that may reflect differences in local agricultural practices, and at the same time, its potential hepatotoxicity also raises concerns about safety [55]. The frequent detection of paroxetine in water bodies suggests the bioaccumulation potential of drugs to affect the safety of the food chain through water pollution [56]. These findings suggest that the differences in contaminants in GELNs likely originate from the local soil and cultivation environment. Therefore, it would be beneficial to develop organic agricultural standards for these contaminants to reduce quality risks. This proposed link, while based on correlative data, highlights a potential approach for quality control. In addition, since these biomarker contaminants do not involve the known exogenous components added during the GELN extraction process, and no known extraction method can remove them, these parameters may also be relevant for ginger nanomaterials obtained by other extraction methods. In brief, six compounds were identified as key quality and safety parameters. Consequently, the relationship between these biomarker contaminants and quality was established. In future research, we will broaden the sample range to obtain more elaborate quality control parameters, enabling a paradigm shift in quality control from post-production testing to proactive, source-level risk management.

### 4.4. The Dual Role of Microbial Metabolism

Prior work showed that ginger–GELNs-co-expressed miRNAs express stress responses [14], and that mycorrhizal symbiosis enhances root and PELN growth under stress [57,58]. In this work, the “microbial metabolism in diverse environments” pathway was enriched in the KEGG enrichment analysis. Geographical variations in the enrichment of plant metabolites within the microbial metabolic network leave a distinct biochemical signature in GELNs, potentially reflecting plant–microbe interaction states and providing a plausible basis for observed quality differences. Among the 26 metabolites enriched in the microbial metabolism in diverse environments pathway, the BA, CL, and JS samples were found to contribute 14, 8, and 4 metabolites, respectively (Figure 5b). This gradient in the influence of microorganisms suggests that the pathway exerts the most substantial impact on BA, with CL coming next, and JS following thereafter. Notably, the order of microbial influence (BA > CL > JS) was inversely correlated with the quality ranking of GELNs (BA < CL < JS). This paradoxical phenomenon suggests that microbial stress does not simply enhance the quality of GELNs. This suggests that microbial stress has a dual impact on GELNs’ quality: while influencing their composition through metabolic pathways, it may also introduce negative effects. The inability of KEGG pathway analysis to identify specific microbial species limits its utility, despite revealing geographical differences in associated metabolites. To clarify the specific microbial species, further research, such as metagenomic analysis of the ginger rhizosphere soil microbial community, is necessary. Moreover, quality markers like mollicellin I correlate with plant KEGG pathway-enriched metabolites. The demonstration provides correlative evidence that GELN quality may be impaired by environmental microorganisms via disruption of plant metabolism. This work opens new research vistas for plant nanovesicles in biological stress, highlighting the critical impact of geographical origin on GELN quality and safety. Future studies should characterize the ginger growth microbiome and elucidate how microbial stresses, such as from *Chaetomium brasiliense*, compromise GELN quality.

### 4.5. Sugar Metabolism Links to GELN Function

JS GELNs showed significant overexpression in both galactose metabolism and starch/sucrose metabolism pathways. Interestingly, JS ginger is affectionately known as ‘sweet ginger’ in its production region. This also indirectly proves that the non-targeted metabolomics analysis method we adopted can accurately analyze the characteristics of the samples. Different GELNs sugar metabolism pathways had preferences for different sugars to be synthesized (D-fructose and melibiose for JS, α-D-glucose for BA, D-mannose for CL). The enhanced sugar metabolism in JS ginger likely promotes GELN biogenesis by supplying both structural precursors for glycoprotein/glycolipid synthesis and the metabolic energy required for vesicle assembly [59,60,61]. This hypothesis is consistent with the observed higher particle concentration at fixed protein levels in JS GELNs, supporting a potential link between sugar metabolism and vesicle yield in our study. Moreover, this metabolic pathway may also influence functionality by shaping a distinct membrane composition that, in synergy with a smaller particle size, underlies the high cellular uptake of JS-GELNs. However, it is not known what causes the glucose metabolism characteristics of JS. Thus, further genetic evidence was deemed necessary to confirm. These findings provide new insights for screening and cultivating ginger cultivars capable of yielding higher-quality GELNs.

## 5. Conclusions

This study demonstrates that the geographic origin of raw ginger plays an important role in shaping the chemical composition and functional properties of GELNs. Untargeted metabolomics revealed 190 metabolites with distinct geographic clustering, underpinning the origin-dependent composition of GELNs. Functional assays confirmed region-specific variations in antioxidant capacity, cellular viability, and internalization efficiency. By integrating OPLS-DA and Spearman correlation analysis, 32 metabolites were identified as significantly associated with antioxidant activity. A robust quality and safety marker panel comprising six key compounds was established through random forest validation: paroxetine, mollicellin I, cypermethrin, and mammea a/ad cyclo d (showing negative correlation with antioxidant activity), along with 1-methylhistidine and defluoroatorvastatin (exhibiting positive correlation). Evaluation using this panel showed that GELNs from the JS origin exhibited the most favorable quality and safety profile, followed by CL and BA. Eight key metabolic pathways contribute to these geographical differences, with “microbial metabolism in diverse environments” and “galactose metabolism” emerging as the most prominent factors. This, coupled with the identification of mollicellin I (from symbiotic organism *Chaetomium brasiliense*) as a quality marker, provides tangible evidence linking the observed quality variation to geography-specific microbiota.

In summary, this study not only establishes a transferable quality control framework but also deciphers the mechanism underlying geographical variations, which is supported by metabolic pathway analysis and specific microbial-derived markers. This work lays a foundation for the objective evaluation of natural food nanomaterials and could pave the way for advancing the field from passive detection to active design. Notwithstanding these contributions, it is important to acknowledge that the level of confidence achievable in metabolite identification without authentic standards represents a methodological constraint. Future studies incorporating such validation will be crucial to further fortify these findings.

## Figures and Tables

**Table 1:** Summary of six key quality markers identified in GELNs by metabolomics.

NO.	Name	Formula	Adduct	VIP	*p* Value	*m*/*z*	rt (s)	Score
1	Mollicellin I	C_21_H_22_O_6_	[M + H] +	2.4526	0.0004	371.16632	361.4120	0.9960
2	Cypermethrin	C_22_H_19_Cl_2_NO_3_	[M + H + NH_3_ + 2i] +	2.4232	0.0098	435.12468	355.4390	0.9727
3	Mammea a/ad cyclo d	C_24_H_22_O_5_	[M + H] +	2.4083	0.0034	391.14851	102.5639	0.9203
4	1-Methylhistidine	C_7_H_11_N_3_O_2_	[M + H − H_2_O] +	2.3441	0.0009	152.08178	87.2475	0.8787
5	Paroxetine	C_19_H_20_FNO_3_	[M + H] +	2.1854	0.0462	330.13245	360.5200	0.8996
6	Defluoroatorvastatin	C_33_H_36_N_2_O_5_	[M − H] −	2.1361	0.0181	539.26373	45.5001	0.8306

## Data Availability

The original contributions presented in this study are included in the article/Supplementary Materials. Further inquiries can be directed to the corresponding authors.

## Supplementary Materials

The following supporting information can be downloaded at: https://www.mdpi.com/article/10.3390/foods15020408/s1, Figure S1: Morphology of gingers from three geographic locations. Figure S2: Overlap of the total ion chromatograms (TICs) of the QC samples. Figure S3: Comprehensive chemical profiling of GELNs. Figure S4: The permutation test *p*-values for Q2 and R2Y in the PLS-DA and OPLS-DA models. Figure S5: Cluster analysis of different lipids’ and lipid-like molecules’ abundances of GELNs from gingers of three geographic locations. Figure S6: Confocal micrographs of Caco-2 cells showing the internalization. Figure S7: Correlation plots between protein content and total abundance of samples, active ginger ingredients (6-gingerol, 8-gingerol, 10-gingerol), and amino acids, peptides, and their analogues. Table S1: The information on ginger samples from different geographical locations. Table S2: The protein content of GELNs from gingers of three geographic origins (*n* = 3, data are mean ±  SD). Table S3: Summary information of 190 differential compounds of exosome-like nanovesicles derived from gingers of three geographic origins after normalization by sample protein concentration (VIP > 1, *p* < 0.05). References [62,63,64,65] are cited in the Supplementary Materials.
