# Supplementary material for: Untargeted Metabolomics Reveals Raw Material Geographic Origin as a Key Factor Shaping the Quality of Ginger-Derived Exosome-like Nanovesicles
Source: Foods. 2026 Jan 22;15(2):408. doi: 10.3390/foods15020408 (PMC12841063; doi:10.3390/foods15020408)
Supplement: Supplementary file 1 [file foods-15-00408-s001.zip › foods-4094084-supplementary.pdf]

Supplementary materials

Untargeted Metabolomics Reveals Raw Material Geographic Origin as a Key Factor Shaping the Quality of Ginger-Derived Exosome-like Nanovesicles

Zhuo Chen [1,2,3,4], Xinyi Zhang [1,2,4], Liuliu Luo [1,2,4], Qiang Liu [4,5], Pingduo Chen [1,2,4], Jinnian Peng [3], Fangfang Min [1,2,4], Yunpeng Shen [1,2,4], Jingjing Li [1,2,4], Yongning Wu [6,7,*] and Hongbing Chen [1,2,4,7,*]

1 State Key Laboratory of Food Science and Resources, Nanchang University, Nanchang 330047, China; 352335920004@email.ncu.edu.cn (Z.C.); 407900240102@email.ncu.edu.cn (X.Z.); 407900240074@ncu.edu.cn (L.L.); 417900230224@ncu.edu.cn (P.C.); min2fang@ncu.edu.cn (F.M.); syp@ncu.edu.cn (Y.S.); 357900230012@email.ncu.edu.cn (J.L.)
2 School of Food Science and Technology, Nanchang University, Nanchang 330031, China
3 School of Pharmacy, Gannan Medical University, Ganzhou 341000, China; pengjinnian2017@gmu.edu.cn
4 Sino-German Joint Research Institute, Nanchang University, Nanchang 330047, China; andyliu@dlut.edu.cn
5 Faculty of Medicine, Dalian University of Technology, Dalian 116024, China
6 NHC Key Lab of Food Safety Risk Assessment, China National Center for Food Safety Risk Assessment (CFSA), Beijing 100022, China
7 Jiangxi Province Key Laboratory of Food Allergy, Nanchang University, Nanchang 330047, China
* Correspondence: wuyongning@cfsa.net.cn (Y.W.); chenhongbing@ncu.edu.cn (H.C.)

1.Supplementary Tables

Table S1. The information of ginger samples from different geographical location.

Ginger name	Jiushan ginger (JS)	Boai ginger (BA)	Chaling ginger (CL)	Reference
Geographical Feature	Xingguo, Jiangxi	Bo'ai, Henan	Chaling, Hunan	[1-3]
Location (Coordinates)	26°20'28.4"N, 115°21'28.7" E	35°05'089"–35°12'129"N, 112°59'115"–113°10'319" E	26°46'59" N, 113°45'0" E	[1-3]
Climate Zone	Humid subtropical monsoon climate	Warm-temperate continental monsoon climate	Humid subtropical monsoon climate	[1-3]
Annual Average Temperature	18.8 °C	14.7 °C	17.9 °C	[1-3]
Annual Precipitation	1074.6 mm	597.1 mm	1370 – 1423 mm	[1-3]
Annual Sunshine Duration	1926.5 h	2432.6 h	1670 – 1718 h	[1-3]
Predominant Soil Type	Red soil, yellow soils, purple soil, paddy soils, and meadow soilsl	Alluvial medium loam	Sandy loam	[1,2,4]
Typical Soil Properties	Acidic, relatively clayey	Deep soil layer, strong water and fertility retention	Loose, fertile, good permeability/drainage	[1,2,4]

Table S2. The protein content of GELNs from gingers of three geographic origins (n = 3, data are mean ± SD).

Ginger name	Sample1 (mg/mL)	Sample2 (mg/mL)	Sample3 (mg/mL)
JS	1.91±0.04	2.73±0.03	3.13±0.07
CL	1.23±0.04	1.07±0.07	0.98±0.03
BA	1.68±0.13	1.52±0.01	1.74±0.04

Table S3. Summary information of 190 Differential compounds of exosome-like nanovesicles derived from gingers of three geographic origins after normalization by sample protein concentration (VIP > 1, $p < 0.05$).

NO.	Name	VIP	JS relative abundance	CL relative abundance	BA relative abundance	adduct	m/z	rt(s)	SubClass
1	Mollicellin i	2.4526	33544812.56 ± 3850895.69	52258252.78± 8543422.03	83903804.95 ± 7999517.26	[M+H] +	371.1663	361.4120	
2	Cypermethrin	2.4232	167905.99 ± 98920.96	610775.02± 148094.40	3269703.58 ± 1506631.32	[M+H+NH$_3$+2i] +	435.1247	355.4390	Fatty acid esters
3	Mammea a/ad cyclo d	2.4083	308100.96 ± 221229.31	1088560.56± 432450.59	12847414.99 ± 5110582.22	[M + H] +	391.1485	102.5639	Prenylated neoflavonoids
4	1-Methylhistidine	2.3441	66328108.93± 1960237.93	31090174.0± 13626321.42	18721653.20± 2642516.02	[M+H-H$_2$O] +	152.0818	87.2475	Amino acids, peptides, and analogues
5	11,12-dihydroxy-13-methoxy-4,5,8-trimethyl-3-(2-methylpropyl)-1h,2h,3h,4h,6ah,9h,10h,11h,12h,13h,14h,15h,15bh-cycloundeca[e]isoindole-1,15-dione	2.3016	4533963.88 ± 900317.03	5951711.43± 1252704.49	20622539.89 ± 5139813.71	[M +Na] +	456.2731	114.0675	Aspochalasins
6	Cytosine	2.1873	355709807.37 ± 66730255.13	519690971.73 ± 108634787.39	665409345.27 ± 66435470.94	[M +Na] +	134.0187	496.8050	Pyrimidines and pyrimidine derivatives
7	Paroxetine	2.1854	1220391.80 ± 589129.03	1649482.97 ± 634490.53	11249173.95 ± 7296247.27	[M+H] +	330.1325	360.5200	Phenylpiperidines
8	Cocaine	2.1426	9486197.82 ± 3598885.52	5661328.64 ± 1552092.53	3468706.70 ± 654649.07	[M+H] +	304.1645	364.511	Benzoic acids and derivatives
9	L-Isoleucine	2.1419	21026366.97 ± 7026476.48	37153256.44 ± 2844522.84	46702314.70 ± 9780759.56	[M-H] -	130.086	522.544	Amino acids, peptides, and analogues
10	Defluoroatorvastatin	2.1361	34549832.53 ± 13781242.77	6812302.19 ± 3295377.47	4602688.92 ± 4701467.94	[M-H] -	539.2637	45.5001	Substituted pyrroles
11	D-mannitol	2.1146	65866386.96 ± 21901320.25	76751929.59 ± 7657242.15	130949222.63 ± 14174967.30	[M-H] -	181.0706	266.3280	Carbohydrates and carbohydrate conjugates
12	Alanine	2.0958	8812886.54 ± 6563686.10	41797597.52 ± 2776200.29	60843920.81 ± 5080278.78	[M+H] +	89.0714	74.4475	Amino acids, peptides, and analogues
13	Glycine	2.0878	12931206.81 ± 4347046.86	16343722.36 ± 2742264.49	30043393.82 ± 8817184.98	[M-H] -	74.0236	515.0710	Amino acids, peptides, and analogues
14	.alpha.-cyperone	2.0829	32480914.22 ± 13416301.77	45539457.42 ± 13553137.83	77027581.03 ± 12404950.48	[M+H-C$_8$H$_{12}$] +	111.0919	44.4350	Sesquiterpenoids
15	Propylene glycol propyl ether	2.0828	17443119.47 ± 471554.44	36248262.34 ± 1971885.71	34344823.70 ± 3279735.52	[M+H-H$_2$O] +	101.1077	370.3030	Alcohols and polyols
16	4-pyridoxic acid	2.0803	11828635.82 ± 5129898.34	18007753.13 ± 4690233.10	32258173.88 ± 11436367.91	[M+H] +	184.0602	57.8949	Pyridinecarboxylic acids and derivatives
17	L-Valine	2.0768	89881443.50 ± 53822077.98	168361883.60 ± 27669831.66	279776712.90 ± 13332507.48	[M-H] -	116.0705	494.5335	Amino acids, peptides, and analogues
18	Alpha-ketoisovaleric acid	2.0704	1235822429.57 ± 909466200.41	1770952985.00 ± 423775077.07	3498422816.67 ± 799459140.94	[M-H] -	115.0388	285.2310	Short-chain keto acids and derivatives
19	4-methylbenzamide oxime	2.0461	15923733.69 ± 4721531.32	33798467.06 ± 4909711.02	43909313.04 ± 16633864.81	[M+H] +	151.0751	166.6770	Toluenes
20	3-methylglutarylcarnitine	2.04	9891005.20 ±	41520476.80 ±	41083123.88 ±	[M-H-C$_7$	145.0	59.6	Fatty acid esters

NO.	Name	VIP	JS relative abundance	CL relative abundance	BA relative abundance	adduct	m/z	rt(s)	SubClass
			3120397.44	7867615.58	15297986.67	$H_{13}NO_2$]-	495	906	
21	Chidamide	2.0242	2174543.00 ± 2915788.28	1624847.49 ± 854451.38	21838536.80 ± 7015267.12	[M+H] +	391.1488	132.5020	Anilides
22	21-carboxylic acid triamcinolone acetonide	2.0056	12544251.27 ± 5241783.41	10340212.67 ± 5261680.44	1362681.34 ± 1236437.10	[M-H] -	447.1649	104.0170	Pregnane steroids
23	Matairesinol	1.9947	18320867.09 ± 1988298.99	7711051.66 ± 10183258.23	1995050.53 ± 1804337.04	[M-H] -	357.1335	65.5691	Tetrahydrofuran lignans
24	D- (+)-mannose	1.9063	245072896.67 ± 88804854.65	794874233.37 ± 188194224.89	665065714.23 ± 55323943.25	[M-H] -	179.037	491.5970	Carbohydrates and carbohydrate conjugates
25	Allyl isothiocyanate	1.8954	245413264.33 ± 109719762.45	841520758.50 ± 269056351.49	737113897.47 ± 86236050.17	[M-H] -	98.0235	364.9120	
26	N.alpha.-methyl-l-lysine	1.8758	28521329.54 ± 13157000.53	28966975.60 ± 2202451.79	59843657.45 ± 4563869.80	[M+H-NH_3] +	144.113	375.1515	Amino acids, peptides, and analogues
27	O-t-butyl-l-serine, methyl ester	1.8741	15607999.13 ± 2540079.77	40154730.17 ± 6994894.17	33826945.29 ± 3886428.44	[M+H-C_4H_8] +	120.0656	85.9686	Amino acids, peptides, and analogues
28	N-(phosphonomethyl)glycine	1.8685	13084547.70 ± 616658.24	29655557.95 ± 10305177.02	26788177.51 ± 1884682.21	[M-H] -	168.0266	564.4450	Amino acids, peptides, and analogues
29	Udp-galactose	1.8471	943844.45 ± 487394.73	1052946.79 ± 35019.59	275647.83 ± 77707.50	[M-H] -	565.0461	621.1660	Pyrimidine nucleotide sugars
30	5-aminovaleric acid	1.8415	118710034.19 ± 24768772.81	125383769.49 ± 36999666.24	210574521.60 ± 18459308.33	[M-H_2O+H] +	100.0761	319.644	Amino acids, peptides, and analogues
31	L-arabinitol	1.8281	18417728.64 ± 4534430.21	33283175.21 ± 7228026.56	31627815.82 ± 3427371.75	[M-H] -	151.0599	364.9465	Carbohydrates and carbohydrate conjugates
32	Parthenolide	1.8199	5671273.57 ± 991693.77	15655149.35 ± 3116274.44	13773568.69 ± 4399634.92	[M+H-H_2O] +	231.1234	1011.8300	Terpene lactones
33	1h-indole-6-carboxamide, n-hydroxy-1-[(4-methoxyphen yl) methyl]-	1.8042	10594008.90 ± 5181366.81	26852877.86 ± 6025794.18	25259557.64 ± 4241829.76	[M-H-C_7H_6O] -	189.0869	524.604	Indolecarboxylic acids and derivatives
34	Isoxaflutole	1.7958	6856250.91 ± 2387225.08	11398071.54 ± 1973095.79	11882247.52 ± 1110852.21	[M+H] +	360.0687	563.5475	Carbonyl compounds
35	Adipic acid	1.7833	20733078.15 ± 5427092.10	53719573.80 ± 8119732.06	46008781.21 ± 12076731.22	[M-H]-	145.0494	350.5875	Fatty acids and conjugates
36	Porphobilinogen	1.7596	2572044.16 ± 426537.54	7798666.44 ± 857786.92	7063941.23 ± 2972666.61	[M+H-NH_3] +	210.0786	354.3740	Amines
37	(+)-camphor	1.7525	246804652.03 ± 31941479.62	345616680.83 ± 106817516.33	96689451.01 ± 20970432.93	[M+H-H_2O] +	135.1167	43.4520	Monoterpenoids
38	11-oxatetradecanoic acid	1.7319	39819813.09 ± 26976464.02	23743127.08 ± 5151928.06	133874876.67 ± 18988210.16	[M-H] -	229.18	83.5425	Fatty acids and conjugates
39	N,n-dimethylsphingosine	1.7307	10658208.89 ± 9010225.01	67913198.13 ± 11798037.40	68822106.14 ± 17337434.84	[M+H] +	328.3199	54.7349	Amines
40	Secbumeton	1.7294	7225698.54 ± 3116246.06	17860558.69 ± 3696935.58	15343667.66 ± 3331896.53	[M+H] +	226.1796	159.9590	1,3,5-triazines
41	Piperonyl sulfoxide	1.7203	4818714.62 ± 1624644.90	19857968.61 ± 8597022.79	14457389.94 ± 3426824.08	[M+Na] +	347.1666	377.0870	
42	Harmine	1.7117	8571406.59 ± 2338486.76	35537612.29 ± 9054068.76	26213733.16 ± 9714851.10	[M+H] +	213.113	278.9350	
43	2-linoleoylglycerol	1.6974	32389388.75 ± 24463097.87	318444204.10 ± 83649997.00	211730851.25 ± 124973548.34	[M+H-H_2O] +	337.2725	366.2540	Lineolic acids and derivatives
44	Leukotriene d4	1.6899	5468215.00 ± 3188093.70	8175728.00 ± 611301.72	881292.82 ± 342835.83	[M-H] -	495.2437	401.6460	Eicosanoids
45	3-hydroxybenzyl alcohol	1.6881	23757454.60 ± 1214507.50	21544223.83 ± 4303936.75	34373647.20 ± 1288282.76	[M+H-H_2O] +	107.0493	395.6680	Benzyl alcohols
46	.beta.-Cyano-L-alanine	1.68	33868067.82 ±	69105921.14 ±	66307307.78 ±	[M+H-H_2	97.04	70.9	

NO.	Name	VIP	JS relative abundance	CL relative abundance	BA relative abundance	adduct	m/z	rt(s)	SubClass
		53	14383031.10	18909773.86	8784591.51	O] +	01	941	
47	Etodolac	1.6793	75922678.66 ± 22729585.96	150161181.40 ± 683216.76	130684118.20 ± 25713391.60	[M-H-CO$_2$] -	242.1753	155.8975	Indolyl carboxylic acids and derivatives
48	Hydroxyphenyllactic acid	1.6532	23191236.92 ± 4977018.76	8289247.64 ± 2636961.23	10413185.76 ± 3949888.88	[M-H-H$_2$O] -	163.0237	437.6870	
49	Pyridalyl	1.6415	22529269.69 ± 6424498.52	50084507.62 ± 3542498.47	39628701.01 ± 3704162.80	[M+H-C$_9$H$_6$Cl$_4$O$_2$] +	204.086	396.4860	Halobenzenes
50	N-ethylbuphedrone	1.6074	6319255.88 ± 1040586.33	21612199.38 ± 8661228.19	14522310.14 ± 470303.09	[M+H-H$_2$O] +	174.1485	37.5620	Carbonyl compounds
51	Epoxytricarballylic acid	1.6052	20863317.91 ± 5667442.18	18179336.67 ± 3142496.42	32492854.85 ± 1953198.87	[M-H] -	189.0029	642.4270	Tricarboxylic acids and derivatives
52	(+)-abscisic acid	1.6034	55335444.61 ± 9355044.57	86041080.27 ± 10590364.95	23491038.86 ± 5983449.35	[M-H-CO$_2$] -	219.1228	374.9740	Sesquiterpenoids
53	3,3'-dimethyl-4,4'-diaminodiphenylmethane	1.5952	40021158.72 ± 14580556.42	115358781.14 ± 34060156.88	92548055.35 ± 32057442.31	[M+H] +	227.1747	123.4145	Diphenylmethanes
54	4-hydroxyphenylethanol	1.5952	13551388.76 ± 322499.07	28883494.77 ± 3035806.22	21995942.11 ± 968043.77	[M+H] +	139.05	350.3785	1-hydroxy-2-unsubstituted benzenoids
55	Guanabenz	1.5943	7452367.46 ± 1202617.01	20928596.49 ± 6726453.07	14920740.57 ± 1019874.66	[M+H-NH$_3$+2i] +	215.9778	31.6648	Halobenzenes
56	Benzoylcholine	1.5939	4566665.91 ± 721280.98	7419789.92 ± 1350179.72	6658050.99 ± 1039454.97	[M] +	208.1327	501.8195	Benzoic acids and derivatives
57	Thiosulfuric acid	1.5922	4152921.96 ± 1070467.43	11541497.12 ± 3633113.85	8347237.86 ± 1083536.77	[M-2H+Na] -	134.9422	456.1870	Non-metal thiosulfates
58	4-hydroxy-l-isoleucine	1.5854	14764330.89 ± 1047450.76	32776210.70 ± 9905444.01	25667667.04 ± 3527642.79	[M+H-CH$_2$O$_2$] +	102.0917	6.1100	Amino acids, peptides, and analogues
59	N-acetylsphingosine	1.5819	4010682.36 ± 722633.56	11100838.86 ± 1075127.26	7702069.28 ± 317047.85	[M+H-2H$_2$O] +	306.278	265.0400	Ceramides
60	Enalaprilat	1.5680	281413436.13 ± 104942330.52	722606137.83 ± 271602327.23	51193747.99 ± 16373597.68	[M-H-H$_2$O] -	329.175	41.8179	Amino acids, peptides, and analogues
61	D-psicose	1.5618	3357318613.33 ± 918985964.32	8461752099.67 ± 1641144797.84	6621174763.33 ± 2119972207.80	[M+Cl] -	215.0319	390.4505	Carbohydrates and carbohydrate conjugates
62	18-hydroxy-5z,8z,11z,14z,16e-eicosapentaenoic acid	1.5550	11355631.64 ± 7985630.05	7258881.14 ± 3968498.95	42993276.18 ± 24276347.92	[M-H-CO$_2$] -	273.2217	99.5099	Eicosanoids
63	Trinexapac-ethyl	1.5533	1438641.82 ± 941413.15	6814281.03 ± 2735180.10	4058591.23 ± 816617.70	[M+Na] +	275.1091	419.1370	
64	1-o-octadecyl-2-o-methyl-sn-glyceryl-3-phosphorylcholine	1.5467	23317536.23 ± 5523114.32	58944563.00 ± 16266041.99	45565888.41 ± 13632759.91	[M+Na] +	546.3989	49.6182	Glycerophosphocholines
65	Monoelaidin	1.5462	11192012.30 ± 7169287.35	99348822.33 ± 19611946.13	55838632.56 ± 32078788.68	[M+H-H$_2$O] +	339.2883	364.8880	Monoradylglycerols
66	[(1r,3r,4r,4as)-4-hydroxy-3,4a,8,8-tetramethyl-4-[2-(5-oxo-2h-furan-3-yl)ethyl]-2,3,5,6,7,8a-hexahydro-1h-naphthalen-1-yl] acetate	1.5462	27597302.05 ± 9716551.94	44774047.10 ± 17753600.48	9866030.42 ± 1180584.38	[M-H] -	377.2169	78.2220	Terpene lactones
67	Saccharopine	1.5374	897598.31 ± 638378.01	434350.04 ± 205622.92	3859208.23 ± 1985265.53	[M+H] +	277.1384	622.6130	Amino acids, peptides, and analogues
68	Linalool oxide	1.5166	432051839.90 ± 147388576.18	587999607.10 ± 70544724.10	209101824.20 ± 64809801.91	[M+H-H$_2$O] +	153.1271	326.6590	
69	1-naphthoic acid	1.5094	116496255.20 ± 6011144.77	415258911.57 ± 129246767.81	265903555.17 ± 64582119.18	[M-H] -	171.0652	71.76890	Naphthalenecarboxylic acids and derivatives
70	Loureirin b	1.5074	26977723.95 ± 4627884.79	65962143.25 ± 14950182.58	47391203.22 ± 4903868.27	[M-H]-	315.1358	37.5378	Chalcones and dihydrochalcones

NO.	Name	VIP	JS relative abundance	CL relative abundance	BA relative abundance	adduct	m/z	rt(s)	SubClass
71	Spirodiclofen	1.4906	5280002.22 ± 1100127.69	10905238.91 ± 2163854.10	8520684.97 ± 1286949.18	[M+H-$C_6H_{10}O$] +	313.0494	554.2950	Halobenzenes
72	Cinobufagin	1.4802	1966785.96 ± 342121.05	8082117.21 ± 2226464.02	4946510.98 ± 1750872.55	[M+H] +	443.2314	290.1340	Steroid lactones
73	L-2-hydroxyglutaric acid	1.4698	143501524.13 ± 34732208.37	309549588.67 ± 63721655.34	235542656.43 ± 42352455.83	[M-H-H_2O] -	129.018	351.0525	Short-chain hydroxy acids and derivatives
74	Perillyl alcohol	1.4546	5576320.77 ± 1345871.98	11716116.85 ± 1796918.91	9058046.34 ± 1540870.51	(M+H-H_2O) +	135.1166	441.5830	
75	Bilirubin	1.4515	850627.25 ± 76854.87	27783373.55 ± 17056688.61	6445828.26 ± 2178487.48	[M+H] +	585.2872	238.6770	Bilirubins
76	5-amino-1,3,3-trimethylcyclohexanemethylamine	1.4465	5153571.26 ± 3041070.24	8903200.99 ± 2032679.70	1683489.47 ± 433548.28	[M+H-NH_3] +	154.1588	331.2500	Cyclohexylamines
77	Indoxyl sulfate	1.4456	7910621.75 ± 1799986.25	23141440.65 ± 8980693.32	15483802.41 ± 2476213.98	[M-H] -	212.0048	33.2390	Arylsulfates
78	Arachidonoyl p-nitroaniline	1.4448	248525117.73 ± 110082646.30	324910657.07 ± 61881528.99	111543115.37 ± 37381073.71	[M+H] +	425.2674	41.3355	Nitrobenzenes
79	Quinate	1.4371	48216809.58 ± 10474996.01	138130745.53 ± 16721964.59	90293535.99 ± 15614607.35	[M-H] -	191.055	350.1355	Alcohols and polyols
80	13,14-epoxyfluprostenol isopropyl ester	1.4353	84867664.82 ± 21429480.86	76129135.29 ± 26097971.64	128717441.70 ± 11429446.07	[M+H-H_2O] +	499.2136	384.9860	
81	[(4e)-7-acetyloxy-6-hydroxy-2-methyl-10-oxo-2,3,6,7,8,9-hexahydrooxecin-3-yl] (e)-but-2-enoate	1.4240	4645639.76 ± 223625.43	25952259.91 ± 9400905.79	12368756.92 ± 561869.19	[M-H] -	325.1475	40.1992	Tricarboxylic acids and derivatives
82	2,3-dihydroxybenzoic acid	1.4230	26507686.93 ± 2661915.07	79748511.13 ± 10866046.10	49760096.18 ± 4474080.71	[M-H] -	153.0293	350.1180	Benzoic acids and derivatives
83	Stearamide	1.4227	8214857.65 ± 1512011.33	19459924.80 ± 2835792.79	13567747.44 ± 1074291.53	[M+H] +	284.2939	57.7622	Carboximidic acids
84	1-palmitoyl-2-linoleoyl-rac-glycerol	1.4222	92869197.73 ± 18771134.22	514804839.23 ± 261472655.28	249738077.77 ± 45258990.41	[M+H-$C_{18}H_{32}O_2$] +	313.2728	46.2124	Lineolic acids and derivatives
85	Muscone	1.4202	13659651.54 ± 3407361.23	78219663.90 ± 41095511.53	37481608.71 ± 7264820.18	[M+H] +	239.2365	45.9780	Carbonyl compounds
86	Cis,cis-9,12-octadecadien-1-ol	1.4189	4023383.26 ± 1740566.03	21053709.82 ± 7622805.72	10515583.33 ± 2161005.92	[M+H-H_2O] +	249.2571	47.8868	Fatty alcohols
87	1-palmitoylglycerol	1.4067	103168740.35 ± 22813211.38	519141645.43 ± 251793659.56	260041652.20 ± 44332723.53	[M+H] +	331.2833	46.2800	Monoradylglycerols
88	1-stearoyl-rac-glycerol	1.3946	142791949.53 ± 48729142.42	715129170.97 ± 243491002.92	362889710.13 ± 95748723.56	[M+H] +	359.3145	48.0509	Monoradylglycerols
89	Triclopyr	1.3909	44278177.01 ± 12753412.83	103224219.23 ± 16794479.68	72879899.59 ± 9065886.79	[M+H+2i] +	257.932	609.4860	Halopyridines
90	Methylguanidine	1.3897	92896495.15 ± 25002110.26	219925600.53 ± 40007808.78	153259113.70 ± 15248709.89	[M+H] +	74.0606	46.7852	Guanidines
91	D-arabinose	1.3837	16110932.51 ± 4984456.96	39762556.14 ± 3739343.01	27091795.57 ± 2968811.05	[M-H] -	149.0595	116.9170	Carbohydrates and carbohydrate conjugates
92	Beta-hydroxybutyrate	1.3797	61955548.02 ± 6640263.11	166058509.27 ± 22919191.13	106728935.41 ± 7123523.33	[M-H] -	103.0388	470.8520	Beta hydroxy acids and derivatives
93	4-quinazolinamine, 2-(hexahydro-4-methyl-1h-1,4-diazepin-1-yl)-6,7-dimethoxy-n-[1-(phenylmethyl)-4-piperidinyl]-	1.3747	11376938.32 ± 4061604.96	26755771.29 ± 7957744.79	18976149.98 ± 1706583.54	[M+H] +	491.2907	29.0542	Benzylpiperidines
94	Glycine, 1,1'-(1,8-dioxo-1,8-octanediyl) bis [glycyl-	1.3732	202338311.17 ± 48883262.25	258501573.10 ± 42847291.96	132681120.50 ± 12278862.81	[M-H-H_2O] -	383.1468	299.5620	Amino acids, peptides, and analogues
95	2-hexenal	1.3678	12936701.62 ± 7706966.25	31232087.13 ± 7807507.71	22142024.59 ± 1159927.26	[M+H] +	99.0921	397.0250	Carbonyl compounds

NO.	Name	VIP	JS relative abundance	CL relative abundance	BA relative abundance	adduct	m/z	rt(s)	SubClass
96	Leucine	1.3668	12016751.32 ± 1695097.19	32042853.55 ± 2993240.98	20515743.90 ± 1276262.77	[M-H] -	130.0243	1005.585	Amino acids, peptides, and analogues
97	Pyrocatechol	1.3638	352738270.87 ± 156233238.44	237632968.97 ± 116818128.28	714676985.37 ± 99496757.53	[M-H] -	109.0394	84.5552	Benzenediols
98	Loxistatin acid	1.3596	19162912.87 ± 7570366.31	58650375.18 ± 5371346.51	37053481.59 ± 11213746.22	[M-H-CO_2] -	269.2116	56.8947	Amino acids, peptides, and analogues
99	Linoelaidic acid	1.3571	2007170.99 ± 730354.48	16656060.21 ± 3573297.23	7445473.30 ± 4290360.04	[M+H-2H_2O] +	245.2257	366.2070	Lineolic acids and derivatives
100	Probucol	1.3511	15414958.68 ± 2858700.58	84368138.91 ± 39627215.73	38510847.24 ± 2903796.61	[M-H-$C_{17}H_{27}SO$] -	236.1045	90.5893	Phenylpropanes
101	(1e,4e)-1,5-bis(4-methoxyphenyl) penta-1,4-dien-3-one	1.3485	38257302.82 ± 9265401.19	93600592.33 ± 28815083.85	64617544.70 ± 8743967.45	[M+H] +	295.1169	44.4350	Anisoles
102	L-carvone	1.3445	6587534.49 ± 1652047.60	37046913.48 ± 12559850.64	16935421.94 ± 3516126.18	[M+H] +	151.0962	66.4398	Monoterpenoids
103	Pidotimod	1.3385	19182773.13 ± 9106254.60	14883466.43 ± 5752210.59	34965565.45 ± 6274796.91	[M+H] +	245.0478	496.8050	Amino acids, peptides, and analogues
104	N-lauroyl-d-erythro-sphinganine	1.3385	50938836.65 ± 13238402.43	115243122.49 ± 21446240.30	80500406.11 ± 6184475.00	[M+H] +	484.471	96.3515	Ceramides
105	Thiorphan	1.3194	29063240.02 ± 6532898.85	67283537.49 ± 10894160.51	47037020.72 ± 10070404.62	[M-H] -	252.0726	32.3532	Amino acids, peptides, and analogues
106	D-Mannose	1.3151	7550654.73 ± 5244005.68	42599316.39 ± 9352930.62	28461141.08 ± 19679710.87	(M+K-2H) -	217.0173	298.5650	Carbohydrates and carbohydrate conjugates
107	2-benzothiazolsulfonic acid	1.3141	45624473.06 ± 5979172.74	131190288.06 ± 36111857.36	81336765.36 ± 14474291.03	[M-H] -	213.9629	31.5096	
108	Captopril	1.3115	8676418.12 ± 1317520.40	23390892.08 ± 3813854.54	14619653.05 ± 1138538.70	[M+H-C_4H_6SO] +	116.053	40.4104	Amino acids, peptides, and analogues
109	7-methylguanine	1.3097	83837342.86 ± 26357868.80	206401818.80 ± 27369006.10	139311775.03 ± 26390090.13	[M+H] +	166.0972	306.207	Purines and purine derivatives
110	Citrinin	1.2987	4854393.15 ± 2502273.08	1730170.36 ± 505470.43	14666469.54 ± 4580722.95	[M+H-H_2O] +	233.0626	434.6405	
111	Theophylline	1.2936	67296414.28 ± 29392399.42	167338969.87 ± 30684222.29	110396107.00 ± 7183920.14	[M-H] -	179.0371	74.6057	Purines and purine derivatives
112	1-heptadecanoyl-sn-glycero-3-phosphocholine	1.2869	22415180.91 ± 8124995.40	62864615.66 ± 6157486.90	7001023.92 ± 3305890.37	[M+H-H_2O] +	492.346	286.3080	Glycerophosphocholines
113	2(1h)-pyridinone	1.2835	6190636.13 ± 901003.12	24446903.53 ± 8269515.14	12748328.81 ± 2078617.48	[M+H] +	96.0448	56.9834	Hydropyridines
114	Echinocystic acid	1.2821	18255277.07 ± 8682916.08	26501981.00 ± 2359464.84	9531174.35 ± 711124.93	[M+Na] +	495.3457	41.8893	Triterpenoids
115	Oxandrolone	1.276	11238853.54 ± 5236858.84	29026623.75 ± 9582872.42	19350436.01 ± 3072376.83	[M+H] +	307.2258	55.8176	Steroid lactones
116	3,4-dimethylbenzaldehyde	1.2729	551445530.13 ± 92685779.34	1237856116.20 ± 337228283.72	851002254.73 ± 59612010.34	[M+H] +	135.0803	44.3541	Benzoyl derivatives
117	3,5-dibromo-l-tyrosine	1.2685	7371884.18 ± 2247032.13	16662138.40 ± 1982086.06	11316387.11 ± 865520.84	[M+H] +	337.8978	670.9245	
118	Isofraxidin	1.2596	63721041.40 ± 27133748.17	189001122.00 ± 20447383.27	116951933.33 ± 33311273.53	[M-H] -	221.0298	493.4150	Hydroxycoumarins
119	6-methoxybenzoxazolin-2(3h)-one	1.2561	13284167.93 ± 2930620.21	34265665.69 ± 4928664.26	21700166.81 ± 3206778.50	[M-H] -	164.0108	349.2240	Benzoxazolones
120	Anhydroecgonine methyl ester	1.2420	18999056.74 ± 159254.03	34372889.08 ± 5244695.29	27212257.60 ± 6932625.38	[M+H-$C_2H_{10}ON$] +	118.0652	328.746	N-alkylpyrrolidines
121	Cyclovirobuxin d	1.2396	7819049.70 ± 2505063.19	17118578.75 ± 2794486.58	11701544.42 ± 213501.27	[M+H-CH_5N] +	372.3459	103.1235	Triterpenoids
122	Mannose	1.2278	34589851.80 ± 5391620.22	101115828.80 ± 28239436.62	59349291.99 ± 6053656.96	[M-H] -	179.037	0.9069	Carbohydrates and carbohy-

NO.	Name	VIP	JS relative abundance	CL relative abundance	BA relative abundance	adduct	m/z	rt(s)	SubClass
									drate conjugates
123	Methyl (1-(cyclohexylmethyl)-1h-indole-3-carbonyl)-l-valinate	1.2241	11231599.20 ± 3043587.67	99810828.16 ± 42103989.86	32940218.03 ± 7181572.23	[M+H] +	371.2263	94.1032	Amino acids, peptides, and analogues
124	2-adamantanone	1.2201	6551357.74 ± 1284003.46	15442524.30 ± 3189345.63	10446713.20 ± 2458217.47	[M+H] +	151.1224	1013.8800	Carbonyl compounds
125	Ethoxyquin	1.2200	13393384.65 ± 2625878.45	32736380.55 ± 4235107.70	20848551.87 ± 2116344.58	[M+H] +	218.1534	1.9288	Quinolones and derivatives
126	(4e,8e)-10-(4-hydroxy-6-methoxy-7-methyl-3-oxo-1h-2-benzofuran-5-yl)-4,8-dimethyldeca-4,8-dienoic acid	1.2166	105833257.72 ± 39265750.02	295239917.00 ± 15415692.36	36069547.74 ± 7486990.53	[M-H] -	387.1781	304.0640	Terpene lactones
127	Methylphosphonic acid	1.2136	3517266447.33 ± 647450542.78	7419265028.67 ± 1818604626.42	5146625092.33 ± 559834890.07	[M-H] -	94.9893	306.5820	Organic phosphonic acids
128	D-mannosamine	1.2128	8452310.03 ± 5343771.36	23293934.10 ± 3672542.47	15012084.84 ± 5224228.78	[M+H] +	180.1015	78.5312	Carbohydrates and carbohydrate conjugates
129	Arachidoyl ethanolamide	1.2125	68463255.34 ± 19885952.44	169231971.37 ± 34862725.13	107547794.61 ± 8840632.78	[M+H] +	356.351	131.7630	Amines
130	1,2,4-benzenetriol	1.2069	49073552.65 ± 12186723.36	93302515.37 ± 12481740.25	69555480.90 ± 8524505.46	[M-H] -	125.0231	392.6625	Benzenetriols and derivatives
131	Tetraethylene glycol	1.206	45618450.26 ± 16015476.63	199961028.97 ± 56721294.53	94789173.00 ± 17655066.30	[M+H] +	195.1223	63.9629	Ethers
132	Disopyramide	1.2048	20068190.22 ± 12116749.98	108017120.24 ± 43122218.40	46443482.49 ± 13849780.33	[M+H] +	340.2582	261.7120	Pheniramines
133	2,4-dichlorobenzoic acid	1.2031	197533108.93 ± 44010741.70	1299318008.73 ± 365620610.92	486328152.47 ± 47458577.56	[M-H+2i] -	190.9565	41.0094	Benzoic acids and derivatives
134	Valeric acid	1.2013	9885716.19 ± 2199150.16	62341394.11 ± 31315904.91	23846878.37 ± 3914923.66	[M-H] -	101.0596	94.7266	Fatty acids and conjugates
135	Arg-Gly-Arg	1.1929	73876830.92 ± 32355232.19	724485104.13 ± 299352031.83	217698724.53 ± 45479969.14	[M+H] +	388.2527	94.8816	Amino acids, peptides, and analogues
136	Aminoadipate	1.1823	13507568.13 ± 6164119.36	42012780.03 ± 11600540.54	23853325.50 ± 4658686.18	[M+H] +	162.0084	497.0725	Amino acids, peptides, and analogues
137	N-myristoylsphinganine	1.1746	531145908.80 ± 138124822.18	1130073504.43 ± 157342394.69	765591298.00 ± 47784744.24	[M+H] +	512.5024	132.2705	Ceramides
138	Oleic acid methyl ester	1.1732	25258576.43 ± 13659742.15	79237522.16 ± 31198185.64	8149602.05 ± 4684511.55	[M+H-CH_4O] +	265.2517	195.4865	Fatty acid esters
139	Fluroxypyr	1.1679	16432917.65 ± 6499203.88	11814740.61 ± 4022621.91	24988572.98 ± 4387741.24	[M+H] +	254.966	457.2160	Halopyridines
140	Acetic acid, 2-(4-chloro-2-methylphenoxy)-	1.1657	11951678.93 ± 2193641.42	64651907.71 ± 7270121.49	26177492.72 ± 3425514.59	[M-H] -	199.0061	42.2946	Phenoxyacetic acid derivatives
141	3-methylxanthine	1.1567	23493882.81 ± 7727082.94	5170895.49 ± 5010663.17	8908102.50 ± 5628813.89	[M+H] +	167.0336	302.6400	Purines and purine derivatives
142	Hexylamine	1.1561	23203194.82 ± 6531755.95	50372804.69 ± 7939975.33	33850534.07 ± 3779490.21	[M+H] +	102.1281	342.6980	Amines
143	1-palmitoyl-2-oleoyl-sn-glycerol	1.156	5974081.76 ± 942915.30	19290329.95 ± 1480574.33	2342918.45 ± 1439377.66	[M+H-H_2O] +	577.5173	116.1830	Diradylglycerols
144	7-demethylsuberosin	1.1558	23248641.62 ± 5251253.36	50554552.54 ± 7042230.51	33696751.86 ± 3366808.83	[M-H] -	229.1072	349.2805	Hydroxycoumarins
145	1,2-dimethylimidazole	1.1556	50255544.89 ± 13372415.42	122659339.17 ± 20446832.42	76534041.49 ± 6906167.91	[M+H] +	97.0764	467.4165	Imidazoles
146	D-myo-inositol-3,4,5,6-tetraphosphate	1.1546	40927596.34 ± 25277302.58	142027284.40 ± 24738132.99	74531157.79 ± 13370921.30	[M-$_2$H] 2-	248.9759	311.4570	Alcohols and polyols
147	Kinoprene	1.1545	229108033.93 ± 62237559.32	426347890.53 ± 29894957.40	127419728.80 ± 24826439.93	[M+H-$C_3H_6O_2$] +	203.1792	43.2314	Sesquiterpenoids

NO.	Name	VIP	JS relative abundance	CL relative abundance	BA relative abundance	adduct	m/z	rt(s)	SubClass
148	Pentadecanoic acid	1.1517	66622174.52 ± 17054599.38	328791624.17 ± 74487284.69	137261353.23 ± 6757156.92	[M-H] -	241.2166	55.9810	Fatty acids and conjugates
149	1,5-hexadien-3-ol	1.1457	6874459.14 ± 2912667.86	4956113.90 ± 687524.82	9469726.04 ± 63168.45	[M+H-H_2O] +	81.0453	84.2209	Alcohols and polyols
150	Laurylguanidine	1.1422	19456783.62 ± 3452190.16	61294531.48 ± 10403710.33	33693091.95 ± 8463076.64	[M+H] +	228.268	292.7720	Guanidines
151	10-deacetylbaccatin iii	1.1389	4371515.94 ± 1256050.81	21592392.04 ± 4601552.41	9427855.68 ± 2724431.43	[M+Na] +	567.2398	366.4810	Diterpenoids
152	Pro-Trp	1.1375	1975725900.33 ± 438840741.74	4448789200.67 ± 697197826.47	2878636819.00 ± 186883317.16	[M+H] +	302.3044	129.9590	Amines
153	Phytanic acid	1.1337	125000891.73 ± 35064261.68	443378705.53 ± 44226722.16	223752219.20 ± 43092435.12	[M-H]-	311.2947	66.5601	Diterpenoids
154	Polygodial	1.1328	1889373366.00 ± 482284228.79	4622539214.67 ± 1217696514.23	2885183163.33 ± 282144078.78	[M+H-$C_6H_{12}O_2$] +	119.0857	44.6301	
155	Patchouli alcohol	1.1255	18121782.48 ± 6858480.62	25790433.81 ± 5734600.22	11162844.28 ± 798657.87	[M+H-$C_6H_{14}O$] +	121.1012	402.7330	Alcohols and polyols
156	Trans-traumatic acid	1.1215	6113311.61 ± 4752597.86	14266357.00 ± 5329965.99	1981085.09 ± 193720.53	[M-H-H_2O]-	209.1173	339.3035	Fatty acids and conjugates
157	Chrysanthemic acid	1.1206	148263770.20 ± 4333122.96	292842194.57 ± 108747215.73	85514257.42 ± 11514398.13	[M-H] -	167.1067	51.2309	Monoterpenoids
158	Galangin	1.1153	8976188.24 ± 2401742.10	23997449.32 ± 6883746.39	14247477.33 ± 2331538.02	[M-H] -	269.0874	1006.2050	Flavones
159	Arcaine	1.1035	5912393.41 ± 3007658.49	12636338.70 ± 1034040.81	8724082.22 ± 1537618.02	[M+H-CH_5N_3] +	114.1027	627.4220	Guanidines
160	Cis-9-palmitoleic acid	1.0999	69269951.80 ± 61347534.71	414174030.10 ± 25351443.73	133928376.60 ± 19983844.49	[M-H] -	253.2168	54.3678	Fatty acids and conjugates
161	4-hydroxyhexenal	1.0981	44578601.81 ± 17596574.64	114778598.42 ± 17444730.48	67697686.69 ± 2622891.25	[M+H-H_2O] +	97.0764	400.9160	Carbonyl compounds
162	Trilostane	1.0941	23530950.79 ± 11645105.33	53442858.15 ± 3952706.78	34426446.59 ± 4642184.02	[M+H-H_2O] +	312.1919	425.8975	Estrane steroids
163	Octadecanoic acid	1.0889	13423858400.33 ± 3342658819.45	33273229599.00 ± 3409339724.34	20400433162.00 ± 4246956569.13	[M-H] -	283.2636	59.3544	Fatty acids and conjugates
164	Butabarbital	1.0864	36563009.87 ± 14267970.45	200260344.73 ± 66980638.06	75834901.64 ± 6716818.73	[M-H] -	211.1331	42.8007	Pyrimidines and pyrimidine derivatives
165	Nudifloramide	1.0862	19426805.98 ± 13904803.18	66272066.02 ± 12189894.59	32924399.21 ± 5389572.90	[M+H] +	153.0899	406.3190	Pyridinecarboxylic acids and derivatives
166	Cnicin	1.0816	7129695.59 ± 3625063.99	2603652.05 ± 1109681.32	16349030.70 ± 5194752.02	[M+Na] +	401.1405	385.7905	Terpene lactones
167	Homogentisic acid	1.0752	26638526.10 ± 3408934.35	66327325.82 ± 20590795.74	40737823.96 ± 6905768.81	[M-H] -	167.045	349.6970	Phenylacetic acids
168	Palmitic acid	1.0744	14424049710.67 ± 3656028539.51	36822121793.00 ± 2440429579.29	21814836515.33 ± 3564899429.32	[M-H] -	255.2323	56.2632	Fatty acids and conjugates
169	Dl-norleucinamide	1.0689	18943807.98 ± 4439469.87	107635373.08 ± 13689386.84	44840651.48 ± 19390318.99	[M+H] +	131.1178	463.3280	Amino acids, peptides, and analogues
170	Quercetin	1.0631	90584270.14 ± 21238930.71	220435325.17 ± 53634979.41	132813260.47 ± 8946017.16	[M-H-$C_8H_6O_3$] -	150.9791	349.2610	Flavones
171	Pimonidazole	1.0568	5427846.27 ± 3130449.63	22493053.20 ± 3075165.87	9955245.73 ± 2367495.89	[M+H] +	255.1221	40.3568	Organic nitro compounds
172	Ferimzone	1.0559	135004729.67 ± 33885391.52	312634469.70 ± 56230506.67	196917433.47 ± 33314664.50	[M+H-$C_6H_9N_3$] +	132.0807	44.1965	Toluenes
173	L-hydroxyarginine	1.0521	45271698.55 ± 14727756.62	96913747.84 ± 21522941.10	64596353.88 ± 8753429.86	[M+H-CH5O2N] +	128.0817	389.6440	Amino acids, peptides, and analogues
174	Stearic acid	1.0505	147637488.97 ± 36892143.34	372808079.97 ± 34702543.78	222736865.63 ± 45922157.05	(M+CH_3COO) -	343.2843	59.4111	Fatty acids and conjugates

NO.	Name	VIP	JS relative abundance	CL relative abundance	BA relative abundance	adduct	m/z	rt(s)	SubClass
175	6.beta.-hydroxyeplerenone	1.0505	19410547.66 ± 12415251.34	4450739.04 ± 117509.06	50854496.19 ± 21152556.65	[M+H] +	431.1874	386.7240	Steroid lactones
176	Isoleucine	1.0478	6009420.67 ± 1064346.38	11535581.49 ± 2484203.57	8470466.54 ± 2241987.26	[M-H] -	130.0861	987.1635	Amino acids, peptides, and analogues
177	Linoleoylglycine	1.0475	12707812.66 ± 6185867.56	49963557.12 ± 8323801.92	4177064.77 ± 1907775.16	[M+H-$C_2H_5O_2N$] +	263.236	156.683	Amino acids, peptides, and analogues
178	Sulfuric acid	1.0448	31213834.00 ± 9150712.23	88454025.24 ± 15469018.98	48099476.44 ± 478712.09	[2M-3H+2Na] -	238.8908	607.0565	Non-metal sulfates
179	Germacrone	1.0425	90761074.89 ± 17540218.83	143180095.37 ± 7933490.87	63021472.17 ± 7808714.98	[M+H] +	219.174	42.7706	Sesquiterpenoids
180	Monolinolenin (9c,12c,15c)	1.0344	3171876.36 ± 703869.13	20417805.97 ± 6725460.74	1033097.94 ± 864967.07	[M+H] +	353.2674	159.7695	Lineolic acids and derivatives
181	4-deoxypyridoxine	1.0306	8816185.16 ± 2712863.15	22446549.01 ± 4682668.37	13198889.04 ± 2045929.61	[M-H-C H_2O] -	122.071	500.9505	Methylpyridines
182	Nootkatone	1.0301	50786205.65 ± 13950652.60	100101710.68 ± 16982169.93	30011093.10 ± 3497799.73	[M+H-H 2O] +	201.1635	42.7413	Sesquiterpenoids
183	N,n'-dicarbobenzyloxy-l-ornithine	1.0261	200958526.14 ± 106053103.91	422020132.23 ± 99211507.07	89884234.72 ± 35370118.49	[M-H] -	399.1416	301.6755	Benzyloxycarbonyls
184	Arginine	1.0258	5246674024.67 ± 2796002912.67	12007256934.00 ± 1757213442.05	7659500804.00 ± 911720827.30	[M+H] +	175.1186	627.4215	Amino acids, peptides, and analogues
185	8z,14z-eicosadienoic acid	1.0097	7747436.51 ± 2249340.96	66187877.83 ± 20284918.93	19557034.88 ± 7659564.47	[M-H] -	307.2634	54.4808	Fatty acids and conjugates
186	Eicosenoic acid	1.0090	21577011.69 ± 5833833.51	201327343.23 ± 75060213.14	57256521.02 ± 25784622.78	[M-H] -	309.2788	57.6775	Fatty acids and conjugates
187	N-(1-amino-3,3-dimethyl-1-oxobu-tan-2-yl)-1-pentyl-1h-indole-3-carboxamide	1.0089	52924361.98 ± 27498704.12	323499214.30 ± 122627914.12	108456182.20 ± 17569782.09	[M+H] +	344.2267	86.0250	Amino acids, peptides, and analogues
188	Ethyl 7-chloro-6-fluoro-4-hydroxyquinoline-3-carboxylate	1.0048	59527949.97 ± 15782926.28	143667374.90 ± 12488902.49	85986967.13 ± 13836840.73	[M-H-$C_3H_4O_2$] -	196.0097	36.5468	Quinolones and derivatives
189	Hexaethylene glycol	1.0030	15830521.46 ± 12103492.73	61422832.73 ± 19387457.34	28096259.80 ± 9914145.04	[M+H] +	283.1742	76.0660	Ethers
190	7-ethyl-10-(4-amino-1-piperidino)carbonyloxycamptothecin	1.0022	7605102.11 ± 1872985.96	14006159.64 ± 3063102.33	4802942.45 ± 835121.70	[M+H] +	519.2399	402.7330	

1. The relative abundance data were normalized to the sample protein concentration to account for variations in sample loading.; 2. The relative abundance data are presented as the mean ± SD (n = 3 biological replicates).

2. **Supplementary Figures**

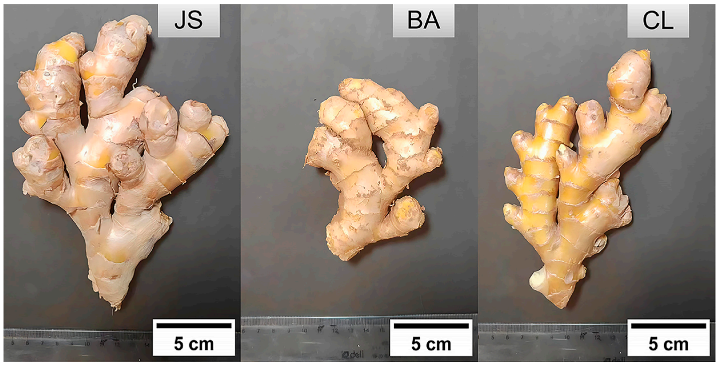

Figure S1. Morphology of gingers from three geographic locations. Distinct morphological differences were exhibited among the gingers from three geographic locations (BA, JS, and CL). Scale bars represent 5 cm.

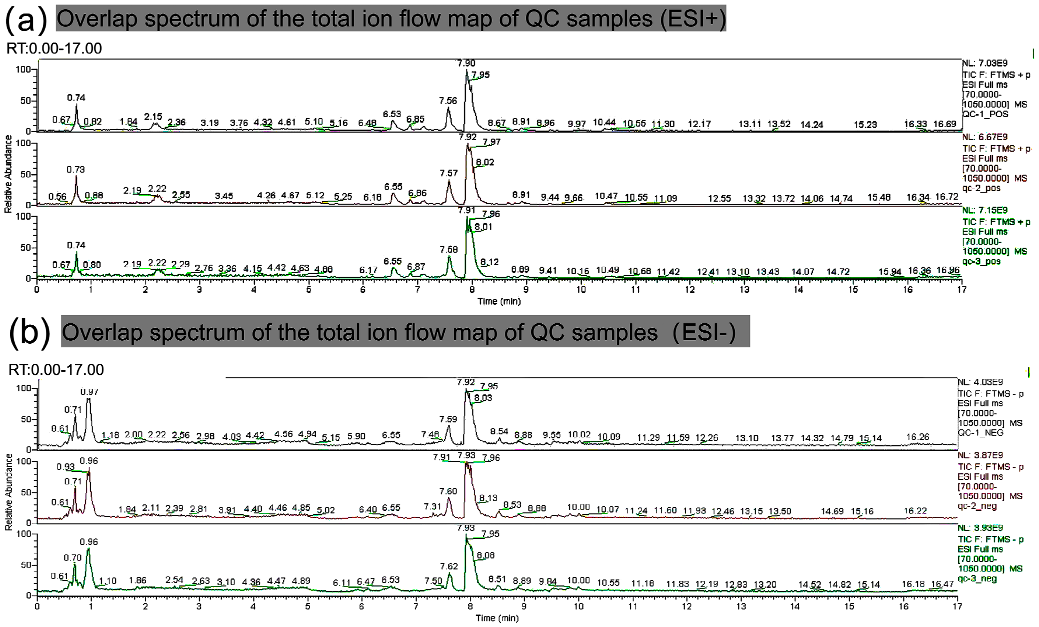

Figure S2. Overlap of the total ion chromatograms (TICs) of the QC samples. The x-axis shows the retention time of each chromatographic peak, and the y-axis represents the intensity values of the peaks. The response intensity and retention time of each peak for the three QC samples largely overlapped in both positive (a) and negative (b) modes.

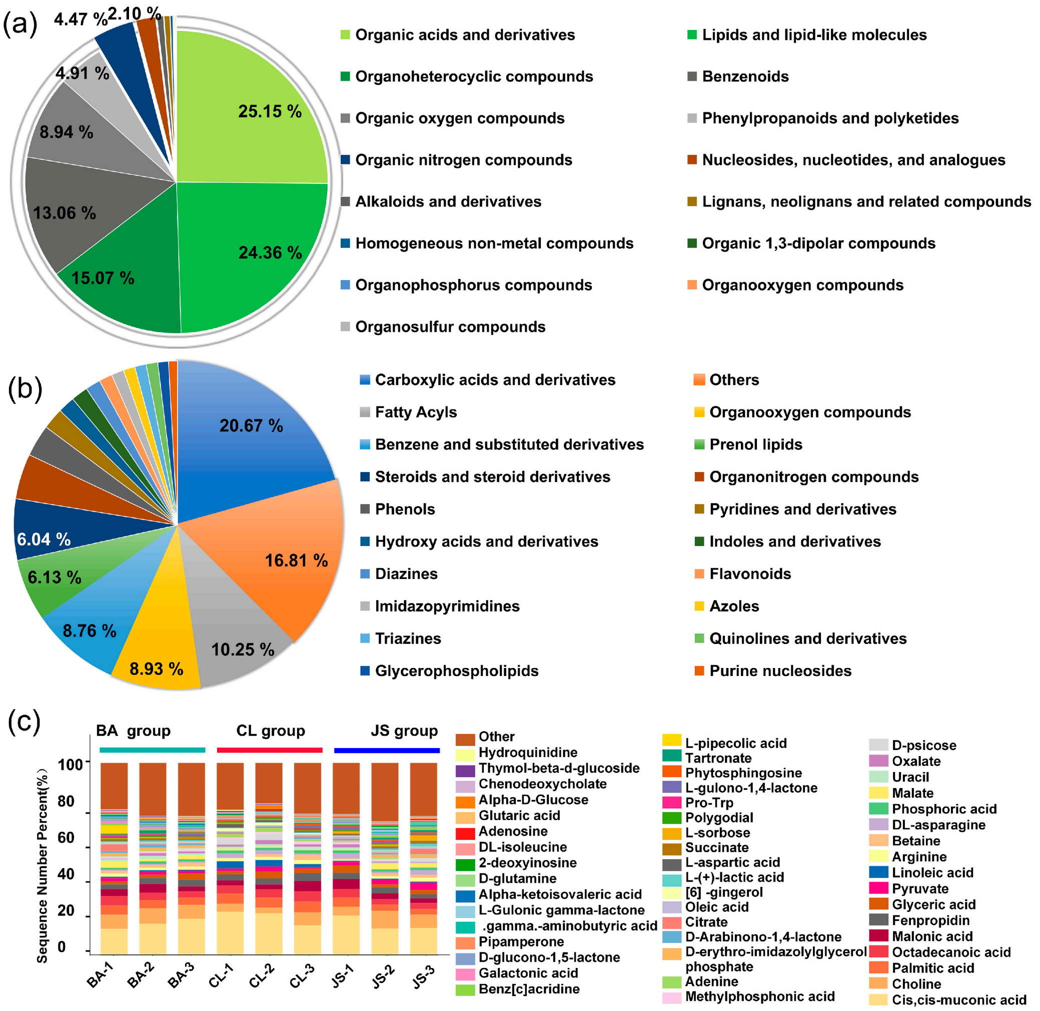

Figure S3. Comprehensive chemical profiling of GELNs. (a) Superclass distribution of identified compounds. (b) Top 20 predominant compound classes. (c) Top 50 high-abundance compounds.

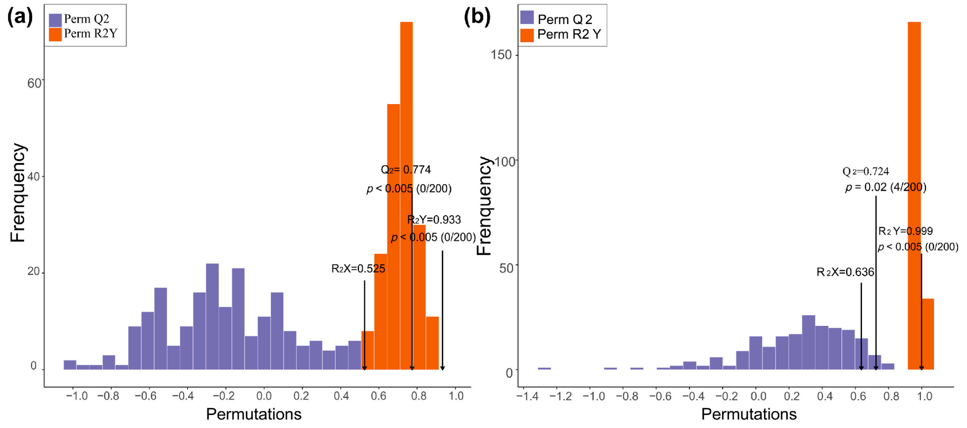

Figure S4. The permutation test *p*-values for Q_2 and R2Y in the PLS-DA and OPLS-DA models. (A) The permutation test *p*-values for Q_2 and R2Y in PLS model. (B)The permutation test p-values for Q2 and R2Y in PLS model in OPLS model.

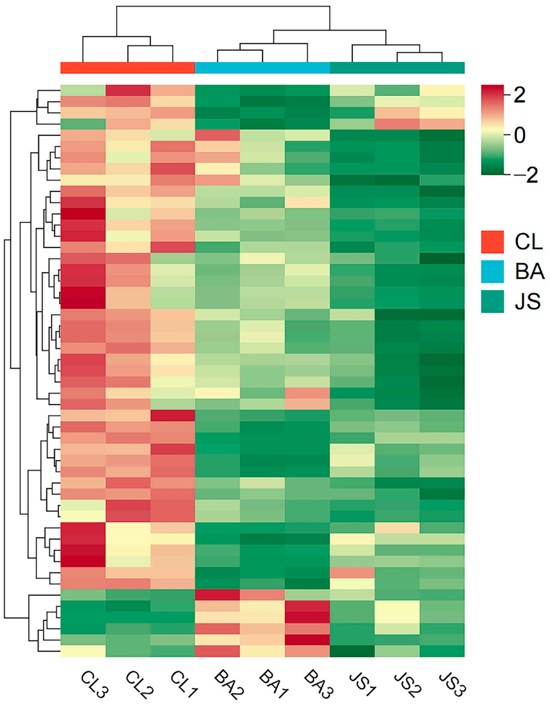

Figure S5. Cluster analysis of difference lipids and lipid-like molecules abundance of the GELNs from gingers of three geographic locations

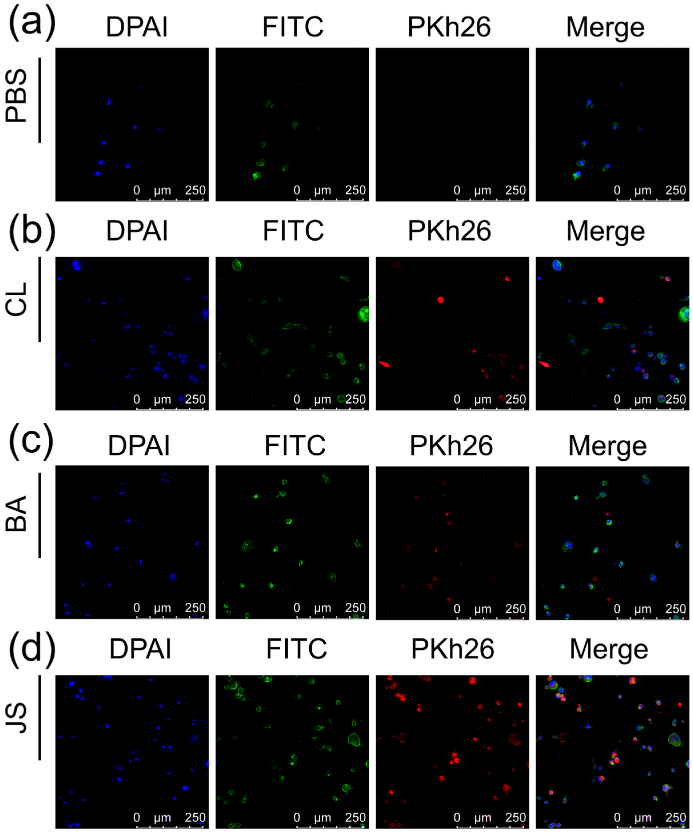

Figure S6. Confocal micrographs of Caco-2 cells showing the internalization. (a) Negative control; (b-d) Confocal fluorescence image of Caco-2 cells internalizing CL GELNs, BA GELNs, and JS GELNs, respectively. Scale bars represent 50 μm.

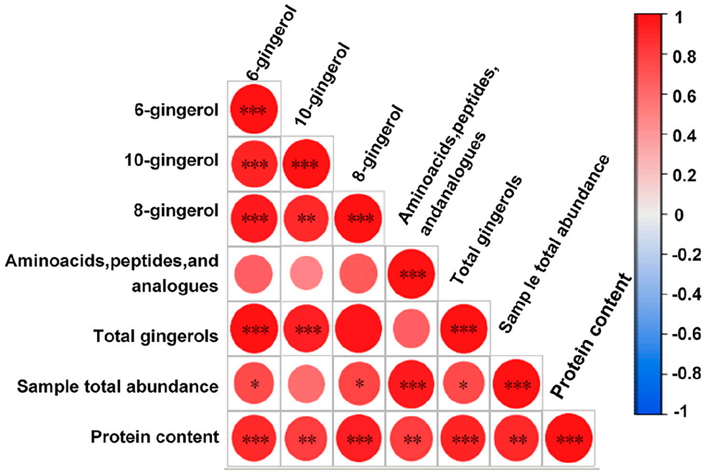

Figure S7. Correlation plots between protein content and total abundance of samples, active ginger ingredients (6-gingerol, 8-gingerol, 10-gingerol), and amino acids, peptides, and their analogues. An asterisk (*) was used to denote statistical significance at $p < 0.05$, a double asterisk (**) was employed to indicate significance at $p < 0.01$, and a triple asterisk (***) was utilized to signify significance at $p < 0.001$.

References

1. National Agricultural Product Geographical Indication Query System. Available online: http://www.anluyun.com/ (accessed on 17 January 2022).
2. Wang, X.; Huang, J.; Yang, L.; Li, Y.; Xia, B.; Li, H.; Deng, X. From Residue to Resource: A Physicochemical and Microbiological Analysis of Soil Microbial Communities through Film Mulch-Enhanced Rice Straw Return Strategies. *Agronomy* **2024**, *14*, 1001, doi:10.3390/agronomy14051001.
3. Li, Y.; Ren, Z.; Zhao, C.; Liang, G. Geographical Origin Traceability of Navel Oranges Based on Near-Infrared Spectroscopy Combined with Deep Learning. *Foods (Basel, Switzerland)* **2025**, *14*, 484, doi:10.3390/foods14030484.
4. Wang, K.; Wang, H.J.; Shi, X.Z.; Weindorf, D.C.; Yu, D.S.; Liang, Y.; Shi, D.M. Landscape analysis of dynamic soil erosion in Subtropical China: A case study in Xingguo County, Jiangxi Province. *Soil and Tillage Research* **2009**, *105*, 313-321, doi:https://doi.org/10.1016/j.still.2008.08.013.
